# Understanding urban inequalities in children’s linear growth outcomes: a trend and decomposition analysis of 39,049 children in Bangladesh (2000-2018)

**DOI:** 10.1186/s12889-021-12181-x

**Published:** 2021-11-30

**Authors:** Hayman Win, Jordyn Wallenborn, Nicole Probst-Hensch, Günther Fink

**Affiliations:** 1 Swiss Tropical and Public Health Institute, Basel, Switzerland; 2 University of Basel, Basel, Switzerland

**Keywords:** Urban health, Health equity, Child nutrition, Stunting, Linear growth, Bangladesh

## Abstract

**Background:**

Despite significant progress in reducing child undernutrition, Bangladesh remains among the top six countries globally with the largest burden of child stunting and has disproportionately high stunting prevalence among the urban poor. We use population representative data to identify key predictors of child stunting in Bangladesh and assess their contributions to linear growth differences observed between urban poor and non-poor children.

**Methods:**

We combined six rounds of Demographic and Health Survey data spanning 2000-2018 and used official poverty rates to classify the urban population into poor and non-poor households. We identified key stunting determinants using stepwise selection method. Regression-decomposition was used to quantify contributions of these key determinants to poverty-based intra-urban differences in child linear growth status.

**Results:**

Key stunting determinants identified in our study predicted 84% of the linear growth difference between urban poor and non-poor children. Child’s place of birth (27%), household wealth (22%), maternal education (18%), and maternal body mass index (11%) were the largest contributors to the intra-urban child linear growth gap. Difference in average height-for-age z score between urban poor and non-poor children declined by 0.31 standard deviations between 2000 and 2018. About one quarter of this observed decrease was explained by reduced differentials between urban poor and non-poor in levels of maternal education and maternal underweight status.

**Conclusions:**

Although the intra-urban disparity in child linear growth status declined over the 2000-2018 period, socioeconomic gaps remain significant. Increased nutrition-sensitive programs and investments targeting the urban poor to improve girls’ education, household food security, and maternal and child health services could aid in further narrowing the remaining linear growth gap.

**Supplementary Information:**

The online version contains supplementary material available at 10.1186/s12889-021-12181-x.

## Background

The Sustainable Development Goals (SDGs) commit all countries to end childhood undernutrition and preventable deaths of children by 2030 [1]. Undernutrition undermines immune system functioning and raises children’s susceptibility to infectious diseases and prolonged illness that can ultimately lead to death [2, 3]. For children that survive, the negative impact of poor nutrition— especially during the period from pregnancy to two-years old—on their ability to grow, learn, and thrive has also been recognized [4, 5].

A key indicator of childhood undernutrition and early life growth faltering is stunting. Children are considered stunted if their height-for-age z score (HAZ) is more than two standard deviations (SD) below the World Health Organization (WHO) child growth standards median [6]. Stunting—resulting from continued exposure to recurrent infections and poor nutritional conditions—is hard to reverse and holds children back from reaching their physical and cognitive potential [7–9]. According to the latest estimates, an estimated 144 million children under-5 are stunted, more than half of whom (78.2 million) live in Asia [10].

One of the most striking features of child undernutrition is the large disparities across and within countries [11]. The global distribution of childhood stunting is concentrated in low-income countries, where large numbers of children generally experience linear growth faltering compared to the international reference median [12]. Within countries, patterns of inequalities in childhood stunting are evident by socioeconomic status [12, 13]. Poorer children are about twice more likely to be stunted than wealthier children [2, 11], and the largest wealth-based gaps in childhood stunting are in South Asia [13]. Geographically, most countries also show an excess in stunting prevalence in rural areas compared to urban areas [2, 11]. However, the ‘urban advantage’ notion has been increasingly challenged in the context of urbanization and growing numbers of urban poor [14–16]. A number of studies examined intra-urban heterogeneities and revealed urban poor and slum residents in some countries to be disadvantaged not only within urban areas, but also when compared with rural populations [17–19].

Our paper focuses on Bangladesh, which has experienced rapid urbanization alongside large intra-urban disparities in child nutritional status. Over the last decades, Bangladesh made remarkable progress to reduce child undernutrition. Between 2000 and 2018, the proportion of stunting among under-five children declined from 51% to 31% [20, 21]. However, Bangladesh remains among the top six countries in the world with the highest burden of child stunting [22], and socioeconomic inequities have accompanied its nutritional progress. The relative inequality in under-five stunting increased by 56% between 1997 and 2014, with the rate of improvement in the richest quintile significantly outpacing that of the poorest [23]*.* Within urban areas, stark residence-based disparities in child nutritional status persist, with half of under-five children in slums found stunted compared to less than one-third in non-slum areas [24].

While the need to address intra-urban inequalities in child nutrition has garnered some policy attention, the empirical evidence explaining these disparities is limited, in part due to scarcity of urban-disaggregated data. With slum-living often viewed to represent concentrated urban poverty [25–27], efforts to understand within-urban differences in child undernutrition have also focused on comparing slum and non-slum residents [19, 28]. However, serial and up-to-date representative urban subpopulation-level data on child nutritional status is limited. Only two rounds of urban health surveys have been conducted so far in Bangladesh [24, 29], with the last one in 2013, and classification of slums in routine household surveys has proved challenging in practice [30, 31]. Therefore, our paper aims to explore poverty-based intra-urban disparity in child linear growth status comparing urban poor and urban non-poor populations based on official urban poverty lines.

## Methods

### Data source

We used data from six Bangladesh Demographic and Health Surveys (BDHS), collected between 2000 and 2018. We excluded the two earliest surveys (1993-94 and 1996-97) due to lack of anthropometric data (1993-94 survey) and a difference in classification of ‘other urban’ areas from subsequent surveys. We included all children aged 0-59 months with valid anthropometry data. Our pooled unweighted data comprised 39,049 live children, of which 12,198 were urban and 26,851 were rural.

### Classification of urban poor

For classifying ‘urban poor,’ we used official poverty head count rates to estimate the percentage of urban people living below the poverty line in the different BDHS survey years. The official poverty estimation in Bangladesh uses the Cost of Basic Needs method. Data for poverty estimates are from Bangladesh Household Income and Expenditure Surveys (HIES) [32]. For each BDHS survey round, we took published upper-line urban poverty head count rates from the nearest survey year of the HIES, and calculated the corresponding cutoff values for urban population in BDHS wealth index scores. We then generated a new binary household variable on urban poverty status to distinguish ‘urban poor’ and ‘urban non-poor’ in the analyses. (See Additional file 1: Appendix 1 for information on urban population below poverty lines and corresponding BDHS asset score cutoff values.)

### Outcome measures

Our primary outcome of interest was stunting, defined as a HAZ of more than two SDs below the 2006 WHO Child Growth Standards median (HAZ<-2). We excluded observations with measurement values that were missing or outside of the WHO-recommended acceptable range (HAZ +/- 6 SDs). We used continuous HAZ variable as a general marker of child linear growth as a secondary outcome.

### Intermediate outcomes

A basic framework developed by UNICEF described disease and poor dietary intake as immediate causes of child undernutrition [33, 34]. To depict patterns in these direct determinants of stunting, we included selected variables of infant and young child feeding (IYCF) practices and recent illness episodes of fever and diarrhea in the descriptive model. However, given the cross-sectional design, we excluded these variables from main regression models due to short-term nature of recent illness episodes and IYCF age-specific focus on children under two years.

### Predictor variables

We included a wide range of household and individual level variables in BDHS to provide a comprehensive profile of urban poor children relative to other socio-economic gradients in the descriptive analysis. The variables were selected based on extant literature and include facets of recognized vulnerabilities among urban poor in Bangladesh. However, as not all variables of interest were collected across BDHS survey years, we limited the main models to include only those available across all rounds and that were applicable to all children under-five. Specifically, we included child’s sex, age, birth order, place of birth, and vaccination status; mother’s age at first marriage, age at first child’s birth, marital status, education, media exposure, numbers of children ever born and living with mother, contraception use, nutritional status, employment, and autonomy; and household wealth, size, head, residence, toilet type, and drinking water source. Antenatal care was available across survey rounds but excluded from main models, as it was limited to only last births. (See Additional file 1: Appendix 2 for details of variable specifications.)

### Statistical analysis

First, we provide descriptive statistics by subgroups of children (rural, urban poor, and non-poor), illustrating key group-level differences. We used Pearson chi-square and Adjusted Wald tests to assess differences across groups. While our paper focuses on within-urban disparities, we included the rural population as a benchmark on poverty; we did not split the rural population into poor and non-poor groups for this purpose, as rural areas tend to be overall poor and disadvantaged relative to urban areas. Second, logistic regression models examined unadjusted and adjusted associations between stunting and key predictors at the national level, as well as disaggregated with urban and rural residence. We then used stepwise selection method with 5% significance threshold to identify the determinants most predictive of stunting in Bangladesh. We focused on key determinants at the national level to maximize statistical power and population variation. Third, we used ordinary least squares (OLS) regression and key stunting determinants (identified in the stepwise selection) to decompose the linear growth difference between urban poor and non-poor children in the pooled urban sample. Contributions of the key stunting determinants to the intra-urban linear growth gap were estimated from the change in OLS estimate of the average HAZ gap with their incremental adjustment (for example, adjusting for the difference in maternal education reduced the predicted HAZ gap from -0.46 to -0.35 SDs, therefore maternal education was estimated to contribute 17.5% of the HAZ gap).

Next, we conducted similar decompositions to assess the sources of change in (i) urban poor and non-poor linear growth gap, and (ii) linear growth status among urban poor children during 2000-2018. To decompose the change in HAZ gap over time, we took the difference of change in each mean variable level among urban poor and non-poor between BDHS rounds 2000 and 2018, and multiplied it by its regression coefficient (estimated association of the factor with HAZ in pooled urban sample). To decompose the change in linear growth status among urban poor children over time, we multiplied the observed change in each mean variable level among urban poor children between 2000 and 2018 by its regression coefficient. We used cluster-robust standard errors in regression estimates to address heteroscedasticity and adjust for survey-level clustering by census enumeration areas. We applied sampling weights in the descriptive analysis; regression analyses were unweighted, as we were primarily interested in survey-level associations. We used statistical software *Stata 15.1* for the analysis [35].

## Results

In total, we analyzed 39,049 child records. Table 1 shows prevalence of stunting and age-specific stunting in our pooled sample, comparing urban poor with rural and urban non-poor children. Across the full sample period (all survey rounds), the proportion of urban poor children stunted was 48.3% (95% CI: 45.5-51.2%), compared to 29.5% (95% CI: 28.0-31.1%) of urban non-poor children and 43.2% (95% CI 42.3-44.2%) of rural children. During early infancy (0-5 months), stunting among urban poor children (16.9%, 95% CI: 12.8-22.0%) was roughly comparable with that of urban non-poor children (16.0%, 95% CI: 13.2-19.2%), and lower than that of rural children (20.9%, 95% CI: 19.2-22.8%). Thereafter, stunting level among urban poor children dramatically increased to 54.5% (95% CI: 49.3-59.6%) during the first two years, compared to 33.6% (95% CI: 30.5-36.8%) among urban non-poor children and 45.9% (95% CI: 44.3-47.5%) among rural children.

**Table 1:** Prevalence of stunting and age-specific stunting in pooled sample comparing rural, urban-poor, and urban non-poor populations (2000-2018)

	Rural		Urban Poor		Urban Non-poor	
	Stunted% (95% CI)	*N*	Stunted% (95% CI)	*N*	Stunted% (95% CI)	*N*
All	43.2 (42.3, 44.2)	30603	48.3 (45.5-51.2)	2207	29.5 (28.0-31.1)	6542
0-5 months	20.9 (19.2, 22.8)	3005	16.9 (12.8, 22.0)	221	16.0 (13.2, 19.2)	617
6-11 months	24.3 (22.5, 26.2)	3195	30.5 (25.0, 36.5)	233	17.3 (14.3, 20.7)	628
12-23 months	45.9 (44.3, 47.5)	6154	54.5 (49.3, 59.6)	463	33.6 (30.5, 36.8)	1365
24-35 months	51.8 (50.0, 53.6)	5953	56.1 (51.6, 60.4)	445	34.7 (31.8, 37.7)	1289
36-47 months	51.5 (49.9, 53.2)	6110	60.4 (55.7, 64.9)	424	32.9 (30.1-35.7)	1313
48-59 months	44.8 (43.1, 46.5)	6186	47.6 (43.2, 52.1)	421	29.1 (26.0-32.3)	1330

Notes: Weighted percentages; standard errors clustered at the survey-cluster level

**Fig. 1:**
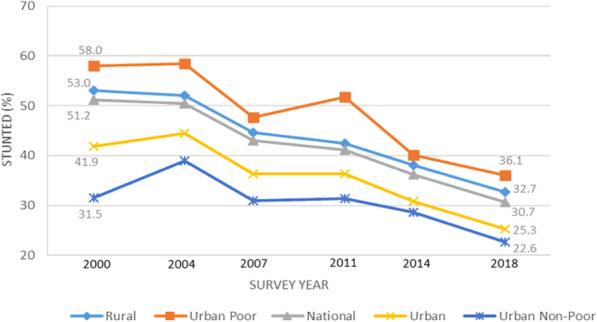
Trends in stunting prevalence by national, urban-rural residence, and urban poverty status (2000-2018)

Figure 1 shows trends in prevalence of child stunting during the 2000-2018 period. Nationally, stunting decreased from 51.2% in 2000 to 30.7% in 2018. The overall decrease in rural areas (20.3 percentage points) outpaced that in urban areas (16.6 percentage points). The trend among urban poor children shows a considerable overall—albeit uneven—drop in stunting (21.9 percentage points), a decrease larger than among rural and urban non-poor (8.9 percentage points) children. However, the prevalence of child stunting was highest among the urban poor at each survey year. The difference in stunting levels between urban poor (36.1%) and non-poor (22.6%) children also remained large in 2018.

Tables 2 and 3 present group-level differences in characteristics of children aged 0-59 months by subgroups of rural, urban poor, and urban non-poor in the pooled sample. Table 2 depicts key proximal determinants of stunting, which center on inadequate diet and recurrent infections. In terms of diet, slightly lower proportions of rural (70.2%) and urban poor (70.1%) breastfed children received minimum meal frequency than urban non-poor (74.6%) breastfed children (*p*=0.008). Less than one third of rural (33.0%) and urban poor (31.8%) children got minimum diet diversity, compared to 44.2% of urban non-poor children (*p*<0.001). While there was no major difference among the groups in exclusive breastfeeding, higher proportions of rural and urban poor children continued breastfeeding after the first year than urban non-poor children (*p*<0.001). In terms of disease, urban poor children seemed more likely to get ill with infections, with 40.4% of urban poor children reporting a recent episode of fever, compared to 32.9% of urban non-poor children and 37.9% of rural children (*p*<0.001).

**Table 2:** Characteristics of proximal stunting determinants (diet and disease) among rural, urban poor, and urban non-poor children (0-59 months) in pooled sample

	Rural	Urban Poor	Urban Non-Poor	*p*-value
**Diet**				
Exclusive breastfeeding (0-5 months)^a^	*N=2515* 69.3%	*N=193* 66.9%	*N=527* 69.1%	0.812
Continued breastfeeding at 1 year (12-15 months**)**^b^	*N=2245* 97.0%	*N=144* 94.6%	*N=467* 90.5%	<0.001
Introducing complementary foods (6-8 months)^c^	*N=1365* 67.6%	*N=102* 66.3%	*N=311* 74.1%	0.093
Minimum meal frequency (breastfed children, 6-23 months)^d^	*N=7552* 70.2%	*N=553* 70.1%	*N=1627* 74.6%	0.008
Minimum dietary diversity (6-23 months)^e^	*N=7973* 33.0%	*N=591* 31.8%	*N=1833* 44.2%	<0.001
**Disease**				
Had fever in last 2 weeks (0-59 months)^f^	*N=30594* 37.9%	*N=2204* 40.4%	*N=6534* 32.9%	<0.001
Had diarrhea in last 2 weeks (6-23 months)^f^	*N=9518* 10.2%	*N=703* 10.8%	*N=2041* 9.2%	0.670

Notes: Weighted percentages; sample includes all children with valid anthropometirc data regardless of living with mother and excludes missing values

^a^Fed only breast milk in the last 24 hours; variable not calculated for 2004 and 2000 waves. ^b^Children 12-15 months fed breast milk the previous day. ^c^Children 6-8 months that ate any solid, semi-solid, or soft foods the previous day; variable not calculated for 2000 wave. ^d^Minimum number of times child received anything to eat (solid, semi-solid, or soft foods) aside from breast milk the previous day (6-8 months: 2 or more times; 9-23 months: 3 or more times); variable not calculated for 2000 wave. ^e^Children 6-23 months that had foods from at least 5 out of 8 food groups the previous day; the food groups are: breastmilk; grains, roots, and tubers; legumes and nuts; dairy products; flesh foods; eggs; vitamin A-rich fruits and vegetables; and other fruits and vegetables. ^f^Child is considered to have had a recent case of fever or diarrhea if the mother reported the illness occurred in last two weeks before survey

**Table 3:** Background characteristics of rural, urban poor, and urban non-poor children (0-59 months) in pooled sample

	Rural	Urban Poor	Urban Non-Poor	*p*-value
**Child Background**				
Male	*N=30603* 51.0%	*N=2207* 51.4%	*N=6542* 51.5%	0.698
Mean age (in months)	*N=30603* 29.4	*N=2207* 28.9	*N=6542* 29.7	0.194
Birth order: First born	*N=30603* 32.7%	*N=2207* 31.1%	*N=6542* 41.3%	<0.001
Place of birth: At home	*N=26286* 82.1%	*N=1931* 84.0%	*N=5276* 48.2%	<0.001
Perceived size at birth: Very small/ smaller than average^a^	*N=13781* 18.4%	*N=970* 22.4%	*N=2732* 15.7%	<0.001
Dewormed in last 6 months (age 6-59 months)^b^	*N=15453* 44.5%	*N=1048* 47.4%	*N=3976* 47.7%	0.025
Vaccinated for measles (0-59 months)^e^	*N=28419* 84.6%	*N=2055* 82.4%	*N=5863* 89.4%	<0.001
Sought medical help when ill with diarrhea^f^	*N=1947* 31.3%	*N=149* 30.8%	*N=373* 50.0%	<0.001
Sought medical help when ill with fever/ cough^f^	*N=13895* 29.9%	*N=1049* 31.3%	*N=2694* 44.8%	<0.001

**Mother’s Background**				
Mean current age (years)	*N=30603* 25.57	*N=2207* 25.26	*N=6542* 26.01	<0.001
Mean age at first marriage (years)	*N=30603* 15.49	*N=2207* 15.10	*N=6542* 16.91	<0.001
Mean age at first child’s birth (years)	*N=30603* 17.71	*N=2207* 17.25	*N=6542* 19.12	<0.001
Divorced/widowed/separated	*N=30603* 1.5%	*N=2207* 2.6%	*N=6542* 1.6%	0.002
Non-Muslim	*N=30599* 8.7%	*N=2207* 9.5%	*N=6540* 8.3%	0.724
Maternal education:	*N=30603*	*N=2207*	*N=6541*	<0.001
No education	26.1%	33.2%	11.2%	
Secondary or higher	43.2%	28.5%	66.4%	
No media exposure	*N=30597* 44.2%	*N=2205* 40.7%	*N=6540* 7.3%	<0.001
Mean number of total children ever born	*N=30603* 2.72	*N=2207* 2.75	*N=6542* 2.17	<0.001
Currently using contraception (any method)	*N=30603* 59.5%	*N=2207* 63.6%	*N=6542* 74.0%	<0.001
4 or more number of children living with mother	*N=30560* 17.2%	*N=2205* 17.4%	*N=6532* 8.2%	<0.001
Very short (<145 cm) maternal stature	*N=30567* 14.2%	*N=2204* 17.9%	*N=6540* 11.4%	<0.001
Underweight (<18.5) Body Mass Index^g^	*N=28850* 31.6%	*N=2087* 33.6%	*N=6306* 14.7%	<0.001
Worked in last 12 months	*N=30603* 24.8%	*N=2207* 32.5%	*N=6541* 21.3%	<0.001
Unskilled laborer^h^	*N=7492* 75.9%	*N=711* 75.5%	*N=1368* 35.9%	<0.001
Low level of autonomy	*N=30349* 37.6%	*N=2174* 33.1%	*N=6473* 26.9%	<0.001
No antenatal care visit (most recent live birth):	*N=22451* 39.6%	*N=1656* 36.6%	*N=4,803* 12.2%	<0.001
No postnatal check within 2 months of birth (most recent live birth):^b^	*N=11616* 45.9%	*N=816* 45.3%	*N=2,984* 28.4%	<0.001

**Household Background**				
Average national wealth index^i^	*N=30603* 2.56	*N=2207* 2.13	*N=6542* 4.60	<0.001
Dhaka Division	*N=30603* 26.7%	*N=2207* 25.9%	*N=6542* 51.4%	<0.001
Average household size	*N=30603* 6.3	*N=2207* 5.7	*N=6542* 6.1	<0.001
Female headed	*N=30603* 9.1%	*N=2207* 6.9%	*N=6542* 8.5%	0.023
Has improved toilet^j^	*N=28369* 41.6%	*N=2067* 40.2%	*N=6131* 70.7%	<0.001
Shares toilet with other households^c^	*N=18021* 36.6%	*N=1345* 52.9%	*N=4746* 44.1%	<0.001
Has improved source of drinking water^j^	*N=28357* 97.1%	*N=2067* 97.9%	*N=6133* 99.6%	<0.001
Has water source located ‘elsewhere’ (not in own dwelling/yard/plot)^b^	*N=16865* 26.9%	*N=1151* 34.2%	*N=2987* 18.9%	<0.001
Has handwashing place observed in dwelling^c^	*N=17096* 91.1%	*N=1,171* 91.1%	*N=4404* 98.6%	<0.001
Cooks with solid fuels^d^	*N=23348* 96.9%	*N=1,698* 98.1%	*N=5496* 40.9%	<0.001

Notes: Weighted means and percentages; sample includes live children with valid anthropometirc data and excludes missing values. Chi-square test (for categorical variables) and Adjusted Wald test (for continuos variables) were used to assess statistical significant differences (p-values) across subgroups

^a^Variable not collected in 2004 and 2018 waves. ^b^Variable not collected in 2000, 2004 and 2007 waves. ^c^Variable not collected in 2000 and 2004 waves. ^d^Variable not collected in 2000 wave. ^e^Children that received measles vaccination at any time before survey, with sources both from immunization card and recall; children 8 months or younger were counted as having received measles vaccination (as the vaccine is typically given only after 9 months of age). ^f^Among children having an episode in last 2 weeks of interview. ^g^Excludes pregnant women. ^h^Among working mothers. ^i^National wealth quintiles scaled from 1 (poorest) to 5 (richest). ^j^Excludes non de-jure residents

Table 3 compares a range of background characteristics among the subgroups. Only 28.5% of urban poor mothers completed schooling at secondary or higher level, compared to 43.2% for rural mothers and 66.4% for urban non-poor mothers (*p*<0.001). More urban poor mothers had very short stature (<145 cm) and were underweight than rural and urban non-poor mothers (*p*<0.001). In terms of maternal and child health seeking, similar proportions of urban poor (84.0%) and rural children (82.1%) were born at home, compared to 48.2% of urban non-poor children (*p*<0.001). A comparable proportion of urban poor and rural mothers, for their last live birth, did not see anyone for antenatal care (36.6% and 39.6%, respectively), compared to 12.2% among urban non-poor mothers (*p*<0.001). On the average national wealth index, urban poor households (2.13) scored slightly lower than the rural households (2.56). Access to an improved toilet was low for both urban poor and rural households (40.2% and 41.6%, respectively), compared to the vast majority of urban non-poor households (70.7%) having this access (*p*<0.001). More urban poor households (52.9%) shared their toilets with other households than rural (36.6%) and urban non-poor (44.1%) households (*p*<0.001). A larger portion of urban poor households (34.2%) did not have piped water into own compound than rural (26.9%) and urban non-poor (18.9%) households (*p*<0.001).

Additional file 1: Appendix 3 shows unadjusted estimates of associations between child stunting and the key variables. Table 4 reports the adjusted estimates. Nationally, being a female child appeared to provide some protection against stunting [OR: 0.92, 95% CI: 0.88-0.97]. Compared to infants (0-11 months), older children (24-59 months) had 3.76 times higher [95% CI: 3.48, 4.07] odds of stunting. Children born at home had 1.28 times higher [95% CI: 1.19, 1.38] odds of stunting than children born in health facilities, and unvaccinated children had 1.22 times higher [95% CI: 1.13, 1.32] odds of stunting than vaccinated children.

**Table 4:** Independent predictors of childhood stunting: adjusted estimates at national level and by urban-rural residence

Stunting OR (95% CI)			
*N (unweighted)*	*National (N=30463)*	*Urban Residence(N=9425)*	*Rural Residence (N=21038)*
*Predictors*			
*Child’s Background*			
**Sex**			
Male	1.00	1.00	1.00
Female	0.92 (0.88,0.97)**	0.89 (0.81,0.98)**	0.94 (0.89,1.00)^
**Age (months)**			
0-11	1.00	1.00	1.00
12-23	3.26 (3.00,3.55)***	3.12 (2.65,3.67)***	3.34 (3.02,3.68)***
24-59	3.76 (3.48,4.07)***	3.17 (2.74,3.67)***	4.07 (3.71,4.47)***
**Birth Order**			
1 to 2	1.00	1.00	1.00
3 or higher	0.89 (0.77,1.02)^	0.97 (0.74,1.28)	0.88 (0.75,1.03)^
**Place of birth**			
Health facility	1.00	1.00	1.00
Home	1.28 (1.19,1.38)***	1.38 (1.22,1.56)***	1.18 (1.07,1.29)***
**Vaccinated (measles)**			
Yes	1.00	1.00	1.00
No	1.22 (1.13,1.32)***	1.34 (1.16,1.54)***	1.17 (1.07,1.29)***

*Mother’s Background*			
**Age at first marriage**			
18 years or older	1.00	1.00	1.00
17 years or younger	1.04 (0.97,1.13)	1.25 (1.08,1.44)**	0.94 (0.86,1.03)
**Age at first child’s birth**			
18 years or older	1.00	1.00	1.00
17 years or younger	1.04 (0.98,1.11)	0.98 (0.87,1.11)	1.06 (0.99,1.14)^
**Highest education level**			
Secondary or higher	1.00	1.00	1.00
No education/ Primary only	1.21 (1.13,1.29)***	1.34 (1.19,1.50)***	1.13 (1.04,1.21)**
**'Media exposure**			
Some	1.00	1.00	1.00
Not at all	1.08 (1.02,1.15)**	0.96 (0.84,1.11)	1.12 (1.05,1.20)***
**Marital status**			
Married	1.00	1.00	1.00
Widowed/divorced/separated	0.95 (0.74,1.23)	1.27 (0.82,1.96)	0.83 (0.61,1.12)
**Total no. children living with mother**			
3 or less	1.00	1.00	1.00
4 or more	1.13 (1.01,1.26)*	1.11 (0.87,1.41)	1.12 (0.99,1.28)^
**Total children ever born**			
1 to 2	1.00	1.00	1.00
3	1.07 (0.93,1.23)	1.00 (0.76,1.30)	1.08 (0.92,1.27)
4 or more	1.18 (0.99,1.40)^	1.28 (0.91,1.80)	1.14 (0.94,1.39)
**Current contraception use**			
Not currently using	1.00	1.00	1.00
Currently using (any method)	0.99 (0.93,1.05)	1.08 (0.96,1.21)	0.96 (0.89,1.02)
**Maternal stature**			
Normal/Tall (155 to <200 cm)	1.00	1.00	1.00
Short (145 to <155 cm)	2.14 (2.00,2.29)***	2.31 (2.03,2.62)***	2.07 (1.92,2.24)***
Very short (<145 cm)	4.70 (4.29,5.14)***	5.38 (4.56,6.35)***	4.44 (3.98,4.95)***
**Body Mass Index**			
Normal (18.5 to <25)	1.00	1.00	1.00
Underweight (<18.5)	1.30 (1.22,1.38)***	1.47 (1.31,1.64)***	1.25 (1.16,1.33)***
Overweight (≥25)	0.71 (0.65,0.78)***	0.74 (0.65,0.86)***	0.71 (0.62,0.80)***
**Worked in last 12 months**			
No	1.00	1.00	1.00
Yes	0.99 (0.92,1.06)	1.06 (0.94,1.19)	0.95 (0.88,1.03)
**Level of autonomy**			
Low	1.00	1.00	1.00
Average-high	0.95 (0.90,1.00)*	0.90 (0.81,1.00)*	0.96 (0.91,1.02)

*Household Background*			
**National Wealth Index**			
Poor	1.00	1.00	1.00
Poorer	0.87 (0.81,0.95)***	0.86 (0.71,1.05)	0.86 (0.78,0.93)***
Middle	0.79 (0.73,0.86)***	0.73 (0.60,0.88)***	0.78 (0.71,0.85)***
Richer	0.69 (0.63,0.76)***	0.68 (0.57,0.83)***	0.63 (0.57,0.71)***
Richest	0.47 (0.42,0.53)***	0.48 (0.39,0.58)***	0.43 (0.37,0.51)***
**Residence**			
Urban	1.00		
Rural	0.93(0.87,1.00)^	-	-
**Division**			
Dhaka	1.00	1.00	1.00
Chittagong	1.07(0.97,1.18)	0.97(0.81,1.15)	1.13 (1.01,1.26)*
Others	0.90 (0.83,0.97)**	0.78 (0.68,0.89)***	1.00(0.88,1.06)
**Household size**			
1 to 6	1.00	1.00	1.00
7 or more	1.07 (1.01,1.14)*	1.03 (0.92,1.15)	1.10 (1.03,1.19)**
**Household head**			
Male	1.00	1.00	1.00
Female	0.93 (0.84,1.02)	0.89 (0.73,1.08)	0.95 (0.84,1.06)
**Type of toilet facility**			
Improved	1.00	1.00	1.00
Non-improved	1.12 (1.05,1.19)***	1.03 (0.93,1.16)	1.15 (1.06,1.24)***
**Source of drinking water**			
Improved	1.00	1.00	1.00
Non-improved	1.24 (1.06,1.44)**	0.80 (0.56,1.16)	1.30 (1.10,1.54)**

Notes: Model accounts for survey fixed effects and standard errors clustered at the survey-cluster level

^*p* ≤0.1; *p ≤0.05; ** *p* ≤0.01; *** *p* ≤0.001

Children of mothers with no or primary level education had 1.21 times higher [95% CI: 1.13, 1.29] odds of stunting than those with mothers having secondary or higher level education. Children of mothers with very short maternal stature (<145cm) had substantially increased odds of child stunting [OR: 4.70, 95% CI: 4.29, 5.14] compared to children of mothers with normal or taller stature (≥155 cm). Compared to children of mothers with normal weight, children of underweight mothers had 1.30 times higher [95% CI: 1.22, 1.38] odds of stunting.

Wealthiest households had 0.47 times [95% CI: 0.42, 0.53] lower odds of child stunting than poorest households. In terms of water and sanitation, households with non-improved source of drinking water had 1.24 times higher [95% CI: 1.06, 1.44] odds of stunting, and households with non-improved toilet types had 1.12 times higher [95% CI: 1.05, 1.19] odds of stunting, compared to households with improved sources.

Additional file 1: Appendix 4 presents the key stunting determinants in resulting from stepwise model selection with 5% significance. All statistically significant predictors in Table 4 survived the search model at the national level. Maternal age at first child’s birth was additionally included among the key determinants.

Table 5 examines estimated contributions of the national-level key stunting determinants (identified from stepwise selection) to the average linear growth gap between urban poor and non-poor children in the pooled urban sample. The first column shows unadjusted average HAZ gap of 0.63 SDs [95% CI: -0.70, -0.56] between urban poor and urban non-poor children. The subsequent columns show incremental adjustments for key covariates, which in combination reduced the intra-urban average HAZ gap from 0.63 SDs to 0.10 SDs [95% CI: -0.24, 0.04] (84.2% net reduction in the HAZ gap). The adjustment for child’s place of birth accounted for the largest reduction (27.0%), followed by household wealth (22.2%), maternal education (17.5%), and maternal body mass index (BMI) (11.1%). Adjustments for child vaccination, household water and sanitation, and maternal reproductive background decreased the average HAZ difference by only 1.6%, 3.2%, and 1.6%, respectively.

**Table 5:** Estimated contributions of key stunting predictors to urban poor and non-poor gap in average HAZ (OLS estimates), pooled urban sample (2000-2018)

*Key stunting predictors*	1 (*N*=12198)	2 (*N*=12198)	3 (*N*=10252)	4 (*N*=10233)	5 (*N*=10231)	6 (*N*=10217)	7 (*N*=10201)	8 (*N*=10194)	9 (*N*=10099)	10 (*N*=9426)	11 (*N*=9426)	12 (*N*=9426)
**Variable’s estimated association with HAZ (95% CI)**												
**Child’s sex** (Female)	--	0.01 (-0.04,0.06)	0.06** (0.01,0.11)	0.06** (0.02,0.11)	0.06* (0.01,0.11)	0.06* (0.01,0.10)	0.06* (0.01,0.10)	0.06* (0.01,0.10)	0.06* (0.01,0.10)	0.05* (0.00,0.10)	0.05* (0.00,0.10)	0.05* (0.00,0.10)
**Child’s age** (12-23 months) (24-59 months)	--	-0.71*** (-0.79,-0.63) -0.78*** (-0.85,-0.71)	-0.72*** (-0.80,-0.64) -0.79*** (-0.86,-0.71)	-0.71*** (-0.79,-0.63) -0.79*** (-0.86,-0.72)	-0.71*** (-0.79,-0.63) -0.78*** (-0.85,-0.71)	-0.70*** (-0.78,-0.62) -0.80*** (-0.88,-0.73)	-0.71*** (-0.79,-0.63) -0.82*** (-0.89,-0.75)	-0.71*** (-0.78,-0.63) -0.81*** (-0.88,-0.74)	-0.71*** (-0.79,-0.63) -0.82*** (-0.89,-0.75)	-0.71*** (-0.79,-0.63) -0.81*** (-0.88,-0.74)	-0.71*** (-0.79,-0.63) -0.81*** (-0.88,-0.74)	-0.71*** (-0.79,-0.63) -0.81*** (-0.88,-0.74)
**Health service use** Child’s place of delivery (at home)	--	--	-0.50*** (-0.56,-0.44)	-0.48*** (-0.54,-0.42)	-0.36*** (-0.42,-0.30)	-0.29*** (-0.35,-0.23)	-0.28*** (-0.33,-0.22)	-0.26*** (-0.31,-0.20)	-0.26*** (-0.31,-0.20)	-0.24*** (-0.30,-0.18)	-0.23*** (-0.30,-0.17)	-0.21*** (-0.27,-0.15)
Child’s vaccination (not vaccinated)	--	--	--	-0.33*** (-0.42,-0.25)	-0.27*** (-0.36,-0.19)	-0.25*** (-0.34,-0.17)	-0.23*** (-0.32,-0.15)	-0.23*** (-0.31,-0.14)	-0.23*** (-0.31,-0.14)	-0.25*** (-0.33,-0.17)	-0.25*** (-0.33,-0.16)	-0.24*** (-0.32,-0.15)
**Maternal education** (no/primary)	--	--	--	--	-0.40*** (-0.46,-0.34)	-0.37*** (-0.43,-0.31)	-0.28*** (-0.34,-0.22)	0.24*** (-0.30,-0.18)	0.25*** (-0.31,-0.19)	0.24*** (-0.30,-0.18)	0.24*** (-0.30,-0.18)	0.21*** (-0.28,-0.15)
**Maternal nutrition** BMI: (underweight) (overweight)	--	--	--	--	--	-0.28*** (-0.34,-0.21) 0.28*** (0.21,0.35)	-0.30*** (-0.36,-0.24) 0.26*** (0.19,0.33)	-0.30*** (-0.36,-0.23) 0.26*** (0.19,0.33)	-0.29*** (-0.35,-0.23) 0.25*** (0.18,0.32)	-0.28*** (-0.34,-0.22) 0.26*** (0.19,0.33)	-0.28*** (-0.35,-0.22) 0.26*** (0.19,0.33)	-0.27*** (-0.34,-0.21) 0.23*** (0.16,0.31)
Stature: (short: 145 to <155cm) (very short: <145 cm)	--	--	--	--	--	--	-0.52*** (-0.57,-0.45) -1.02*** (-1.10,-0.93)	-0.51*** (-0.57,-0.45) -1.02*** (-1.10,-0.93)	-0.51*** (-0.57,-0.45) -1.02*** (-1.10,-0.94)	-0.50*** (-0.56,-0.44) -1.02*** (-1.11,-0.93)	-0.50*** (-0.56,-0.44) -1.02*** (-1.11,-0.93)	-0.50*** (-0.56,-0.44) -1.02*** (-1.10,-0.93)
**Mat. reproductive background** Age at first child’s birth (≤17 years) No. children living with mother (≥4)	--	--	--	--	--	--	--	-0.09*** (-0.14,-0.03)	-0.08** (-0.13,-0.03)	-0.08** (-0.13,-0.02)	-0.08** (-0.14,-0.03)	-0.07** (-0.13,-0.02)
	--	--	--	--	--	--	--	-0.15*** (-0.23,-0.06)	-0.14** (-0.23,-0.05)	-0.15** (-0.24,-0.06)	-0.14** (-0.23,-0.05)	-0.14** (-0.23,-0.05)
**Mat. autonomy & media exposure** Media exposure (not at all) Autonomy level (average-high)	--	--	--	--	--	--	--	--	0.01 (-0.07,0.09)	-0.01 (-0.09,0.07)	-0.01 (-0.09,0.07)	0.03 (0.06,0.11)
	--	--	--	--	--	--	--	--	0.07** (0.02,0.13)	0.07** (0.02,0.13)	0.07** (0.02,0.13)	0.07* (0.01,0.13)
**Household WASH** Toilet type (non-improved) Drinking water source (non-improved)	--	--	--	--	--	--	--	--	--	-0.07* (-0.13,-0.01)	-0.06^ (-0.13,0.01)	-0.03 (-0.09,0.04)
	--	--	--	--	--	--	--	--	--	0.21^ (-0.03,0.44)	0.21^ (-0.03,0.45)	0.22^ (-0.01,0.45)
**Household Division** Chittagong Others	--	--	--	--	--	--	--	--	--	--	-0.06 (0.15,0.04) 0.07^ (0.01,0.14)	-0.04 (-0.14,0.06) 0.10* (0.02,0.17)
**Household SES** Household size (≥7 members) Household wealth (Poorer) (Middle) (Richer) (Richest)	-- --	-- --	-- --	-- --	-- --	-- --	-- --	-- --	-- --	-- --	-- --	0.00 (-0.07,0.06) 0.16**(0.04,0.28) 0.19**(0.06,0.31) 0.17^(-0.00,0.35) 0.37***(0.19,0.57)
**Predicted average HAZ gap (95% CI)**	-0.63*** (-0.70,-0.56)	-0.64*** (-0.71,-0.58)	-0.47*** (-0.54,-0.41)	-0.46*** (-0.53,-0.39)	-0.35*** (-0.42,-0.28)	-0.28*** (-0.35,-0.22)	-0.26*** (-0.32,-0.19)	-0.25*** (-0.31,-0.18)	-0.25*** (-0.32,-0.18)	-0.23*** (-0.30,-0.15)	-0.24*** (-0.31,-0.17)	-0.10^ (-0.24,0.04)
**Variables’ contribution to average HAZ gap**	100%	+1.6%	-27.0%	-1.6%	-17.5%	-11.1%	-3.2%	-1.6%	0.0%	-3.2%	+1.6%	-22.2%
**Total remaining gap**		101.6%	74.6%	73.0%	55.5%	44.4%	41.2%	39.6%	39.6%	36.4%	38%	15.8%

Notes: HAZ: height-for-age z-score; OLS: Ordinary Least Squares; N: total number of observations in pooled urban sample after adjusting for covariates in respective columns; model accounts for survey year fixed effects and standard errors clustered at the survey-cluster level; ^*p* ≤0.1; *p ≤0.05; ** *p* ≤0.01; *** *p* ≤0.001

Columns show incremental adjustments for key stunting determinants identified from stepwise selection with 5% significance and resulting changes in predicted average HAZ gap: Column 1 shows unadjusted predicted average HAZ gap by urban poverty status; column 2 additionally adjusts for child’s demographics (sex and age); columns 3-4 additionally adjusts for health service use (child’s place of delivery and vaccination status); column 5 additionally adjusts for maternal education; columns 6-7 additionally adjust for maternal nutrition (BMI and stature); column 8 additionally adjusts for maternal reproductive background (age at first child’s birth and number of children); column 9 additionally adjusts for mother’s level of autonomy and media exposure; column 10 additionally adjusts for household water and sanitation; column 11 additionally adjusts for household divisional location; and column 12 additionally adjusts for household size and wealth

Additional file 1: Appendix 5 reports logistic regression estimates of the key variables’ effects on stunting differential between urban poor and non-poor children. The results roughly mirror the estimates for the average HAZ gap in Table 5.

Table 6 shows estimated changes in urban poor and non-poor HAZ gap due to changes in difference of mean variable levels among urban poor and non-poor children between 2000 and 2018. During this period, the average HAZ gap between urban poor and non-poor children declined from 0.75 SDs in 2000 (baseline difference in mean HAZ between urban poor and non-poor children) to 0.44 SDs in 2018 (endline difference in mean HAZ between urban poor and non-poor children). The HAZ improved 0.31 SDs on average more for urban poor children than for urban non-poor children. Our key covariates explained 55.2% of this observed decrease in HAZ gap. Of the part explained, the key factors that aided the catch-up of urban poor children were the larger improvements for the urban poor in the areas of maternal education (14.8%), maternal BMI (net of 11.3%), and child vaccination (7.7%). Shifts in child’s age demographics, with higher drops in share of children in older age groups among the urban poor than the non-poor—reflecting increased younger age profile of urban poor children—also accounted for 7.7% of the narrowed average HAZ gap. The changes in household wealth quintiles further contributed a net of 5.5% to the decrease in HAZ gap. In terms of maternal BMI, however, the increase in proportion of overweight mothers was 4.1 percentage points higher among the urban non-poor than among the urban poor, which slightly widened the HAZ gap by 3.2%. The decline in home births was also 6.0 percentage points more among the urban non-poor than among the urban poor and widened the HAZ gap by 4.2%.

**Table 6:** Estimated contributions of change in difference of urban poor and non-poor mean variable levels overtime to change in HAZ gap (2000-2018)

	1	2	3	4	5	6	7	8	9	10
Variable	Baseline levels among urban poor, 2000 (%)	Baseline levels among urban non-poor, 2000 (%)	Endline levels among urban poor, 2018 (%)	Endline levels among urban non-poor, 2018 (%)	Variable change among urban poor, (% points, 2018-2000)	Variable change among urban non-poor (% points, 2018-2000)	Difference in urban poor and non-poor variable change (% points)	Estimated association of factor with HAZ in pooled urban sample	Predicted change in HAZ gap due to difference in urban poor and non-poor variable change (7*8)	Contribution of difference in urban poor and non-poor variable change to difference in urban poor and non-poor HAZ gap change [0.31 SD] (2000-2018) (%)
N (weighted)	348	544	424	1647						
Mean HAZ (observed)	-2.17(SD)	-1.42 (SD)	-1.49 (SD)	-1.05 (SD)	0.68 (SD)	0.37 (SD)	0.31 (SD)			100%
Child’s age (months)										
0-11	19.75	18.44	22.79	19.84	3.04	1.40	1.64	Ref.	-	-
12-23	22.16	20.53	20.74	19.86	-1.42	-0.67	-0.75	-0.714***	0.005	1.61
24-59	58.08	61.03	56.48	60.29	-1.60	-0.74	-2.34	-0.810***	0.019	6.13
Child’s sex (female)	49.41	46.96	48.08	49.27	-1.33	2.31	-3.64	0.054*	-0.002	-0.63
Maternal education (no/ primary education)	88.23	40.89	53.57	27.88	-34.66	-13.01	-21.65	-0.213***	0.046	14.84
Maternal autonomy level (low level)	39.52	28.60	23.52	19.52	-16.00	-9.08	-6.92	-0.071*	0.005	1.61
Maternal media exposure (not at all)	34.69	9.29	52.71	9.71	18.02	0.42	17.60	0.026	0.005	1.61
Maternal BMI										
Normal	53.56	61.29	61.50	48.71	7.94	-12.58	20.52	Ref.	-	-
Underweight	44.25	19.56	17.98	9.74	-26.27	-9.82	-16.45	-0.272***	0.045	14.52
Overweight	2.19	19.15	20.52	41.56	18.33	22.41	-4.08	0.234***	-0.010	-3.23
Maternal Stature										
Normal/tall (155-<200cm)	17.26	23.55	16.39	23.95	-0.87	0.40	-1.27	Ref.	-	-
Short (<155cm)	62.19	63.67	66.69	64.72	4.50	1.05	3.45	-0.497***	-0.017	-5.48
Very short (<145cm)	20.55	12.78	16.92	11.33	-3.63	-1.45	-2.18	-1.016***	0.022	7.10
Maternal age at first child’s birth (≤ 17 years)	65.30	38.17	50.45	32.81	-14.85	-5.36	-9.49	-0.071**	0.007	2.26
No. children living with mother (≥4)	21.80	15.84	10.18	5.01	-11.62	-10.83	-0.79	-0.142**	0.001	0.32
Child’s place of delivery (home)	92.97	61.52	66.59	29.10	-26.38	-32.42	6.04	-0.209***	-0.013	-4.19
Child not vaccinated (measles)	24.95	12.13	11.54	8.57	-13.41	-3.56	-9.85	-0.239***	0.024	7.74
Household toilet type (not improved)	62.89	28.21	49.72	33.14	-13.17	4.93	-18.1	-0.030	0.005	1.61
Household drinking water source (not improved)	1.71	0.69	3.13	0.56	1.42	-0.13	1.55	0.222^	0.003	0.97
Division										
Others	41.27	29.84	54.40	30.49	13.13	0.65	12.48	Ref.	-	-
Dhaka	29.69	49.56	14.73	52.56	-14.96	3.00	-17.96	-0.096*	0.017	5.48
Chittagong	29.04	20.59	30.87	16.95	1.83	-3.64	5.47	-0.132**	-0.007	-2.26
Household size (≥7 members)	29.91	46.72	25.99	26.61	-3.92	-20.11	16.19	-0.003	-0.001	-0.32
National wealth index										
Richest	-	87.34	-	55.13	-	-32.21	32.21	Ref.	-	-
Richer	19.11	12.66	-	35.53	-19.11	22.87	-41.98	-0.205***	0.086	27.74
Middle	34.57	-	23.57	9.34	-11.00	9.34	-20.34	-0.192**	0.039	12.58
Poorer	28.31	-	32.07	-	3.76	-	3.76	-0.221**	-0.008	-2.58
Poorest	18.01	-	44.36	-	26.35	-	26.35	-0.379***	-0.100	-32.26
Total									0.171	55.19%

Notes: HAZ: height-for-age z-score; SD: standard deviation; ^*p* ≤0.1; *p ≤0.05; ** *p* ≤0.01; *** *p* ≤0.001

Columns 5 and 6 show the percentage point changes in mean variable levels among urban poor and non-poor, respectively, during this period. The predicted change in HAZ gap, due to the change in mean variable levels, in column 9 was obtained by multiplying the difference in urban poor and non-poor variable change (column 7) with the relevant factor coefficient from Table 5 (column 8). Column 10 shows the predicted change in HAZ gap, due to the gap change in each variable, as percentage share of the total observed change in HAZ gap (0.31 SDs)

Table 7 further assesses the contributions of key determinants to the observed overall linear growth increase among urban poor children during the 2000-2018 period. Average HAZ among urban poor children increased 0.68 SDs, from -2.17 SDs in 2000 to -1.49 SDs in 2018. Our key covariates on net explained about half of this observed HAZ improvement among urban poor children. The largest part of the explained improvement was due to overall changes in maternal BMI, maternal education, and institutional births. The changes in maternal BMI –both the decrease in share of underweight mothers and the smaller increase in share of overweight mothers among urban poor—accounted for 16.9% of the observed average HAZ improvement. The reduction among urban poor in proportion of mothers with no or only primary level education contributed 10.9% to the linear growth improvement. Improvement in child’s institutional birth and vaccination coverage accounted for 8.1% and 4.7%, respectively, of the linear growth increment. Declines in shares of mothers with four or more children and mothers below 17 years at first child’s birth among the urban poor contributed 4.1% to the average HAZ improvement. The increased proportion of infants among urban poor children, and corresponding decline in share of children in older age groups, contributed 3.4% to linear growth improvement. Overall changes in water and sanitation facilities for urban poor during this period contributed only 1% to the HAZ increase. Shifts in relative wealth accumulation—with the share of urban poor households in the poorest national wealth quintile more than doubling during this period—reversed the expected linear growth improvement for urban poor children by a net of 7.1% during this period.

**Table 7:** Estimated contributions of change in mean variable levels overtime to linear growth change among urban poor (2000-2018)

	1	2	3	4	5	6
Variable	Estimated association of factor with HAZ in pooled urban sample	Baseline levels among urban poor, 2000 (%)	Endline levels among urban poor, 2018 (%)	Observed change in variable (2000-2018), % points (3)-(2)/100	Predicted change in HAZ due to variable change (1)*(4)	Variable’s contribution to observed change in HAZ (%) [0.68]
N (weighted)		348	424			
Mean HAZ (outcome)		-2.17 (SD)	-1.49 (SD)	0.68 (SD)		100%
Child sex (female)	0.054*	49.41	48.08	-0.013	-0.001	-0.15
Child’s age						
0-11 months	Ref.	19.75	22.79	0.030	-	-
12-23 months	-0.714***	22.16	20.74	-0.014	0.010	1.47
24-59 months	-0.810***	58.08	56.48	-0.016	0.013	1.91
Maternal education (no/ primary edu.)	-0.213***	88.23	53.57	-0.347	0.074	10.88
Maternal media exposure (not at all)	0.026	34.69	52.71	0.180	0.005	0.74
Maternal autonomy level (low)	-0.071*	39.52	23.52	-0.160	0.011	1.62
Maternal BMI						
Normal	Ref.	53.56	61.50	0.079	-	-
Underweight	-0.272***	44.25	17.98	-0.263	0.072	10.59
Overweight	0.234***	2.19	20.52	0.183	0.043	6.32
Maternal Stature						
Normal/tall (155-<200cm)	Ref.	17.26	16.39	-0.009	-	-
Short (145 to<155cm)	-0.497***	62.19	66.69	0.045	-0.022	-3.24
Very short (<145cm)	-1.016***	20.55	16.92	-0.036	0.037	5.44
Maternal age at first child’s birth (≤17 yrs)	-0.071**	65.30	50.45	-0.149	0.011	1.62
No. children living with mother (≥4)	-0.142**	21.80	10.18	-0.116	0.017	2.50
Child’s place of delivery (home)	-0.209***	92.97	66.59	-0.264	0.055	8.09
Child not vaccinated (measles)	-0.239***	24.95	11.54	-0.134	0.032	4.71
Household toilet type (not improved)	-0.030	62.89	49.72	-0.132	0.004	0.59
Household water source (not improved)	0.222^	1.71	3.13	0.014	0.003	0.44
Division						
Others	Ref.	41.27	54.40	0.131	-	-
Dhaka	-0.096*	29.69	14.73	-0.150	0.014	2.06
Chittagong	-0.132**	29.04	30.87	0.018	-0.002	-0.29
Household size (≥7 members)	-0.003	29.91	25.99	-0.039	0.000	0.00
National Wealth Index						
Richest	Ref.	-	-	-	-	-
Richer	-0.205***	19.11	-	-0.191	0.039	5.74
Middle	-0.192**	34.57	23.57	-0.110	0.021	3.09
Poorer	-0.221**	28.31	32.07	0.038	-0.008	-1.18
Poorest	-0.379***	18.01	44.36	0.264	-0.100	-14.71
Total					0.328	48.24

Notes: HAZ: height-for-age z score; SD: standard deviation; ^p ≤0.1; *p ≤0.05; ** p ≤0.01; *** p ≤0.001

Column 4 shows the changes in mean variable levels among urban poor between 2000 and 2018. The predicted change in HAZ due to the change in mean variable levels among urban poor children (in column 5) was obtained by multiplying the change in variable (column 4) with its factor coefficient from Table 5 (column 1). Column 6 shows the predicted change in HAZ due to the change in mean variable levels among urban poor children as percentage share of the total observed HAZ change (0.68 SDs)

## Discussion

Despite overall global reductions in childhood stunting, urban inequalities remain large in many settings. Our results from Bangladesh suggest that, during 2000-2018, larger reductions in stunting were achieved among urban poor children than among urban non-poor and rural children. Nevertheless, stunting prevalence remained consistently higher among urban poor children than for urban non-poor children, and also than for rural children.

We identified child demographics (age and sex), health service access (vaccination and institutional delivery), maternal education, maternal nutrition (BMI and stature), exposure to the media, family planning (number of children at home), and household wealth, size, and environment (divisional location and sanitation infrastructure) to be predictive of childhood stunting in Bangladesh. In terms of their absolute magnitude, child’s age group, maternal stature, and household wealth were found to be most important. Stunting rates increase with children’s age [28, 36], given cumulative exposure to poor nutritional conditions over time. Maternal stature likely reflects both genetics and mother’s exposure to poor nutrition in early life [37, 38]. Household wealth has been well established as an important determinant in child undernutrition [38].

Our key stunting determinants at the national level (child’s age, sex, place of birth, and vaccination status; maternal education, BMI, stature, media exposure, and number of children; and household wealth, size, divisional location, and sanitation infrastructure) predicted 84% of the linear growth difference between urban poor and non-poor children. We found that a child’s place of birth, maternal education, maternal BMI, and household wealth were key drivers of the linear growth gap between urban poor and non-poor children. Among the explained factors contributing to decreased intra-urban average HAZ gap between 2000 and 2018, we found that larger absolute improvements for the urban poor than for the urban non-poor in levels of maternal education and maternal BMI (reduction in underweight mothers) were the most important. Changes in relative household wealth played an overall positive but a less prominent role, while progress in child’s institutional birth was greater among the urban non-poor than among the urban poor and thus widened the average HAZ gap. In terms of factors explaining the sizeable linear growth gain among urban poor children between 2000 and 2018, we found that changes in maternal BMI, maternal education, and child’s place of birth were the most important contributors. Shifts in relative wealth status, with an increased share of urban poor households in the poorest national wealth quintile, notably reduced the expected growth improvement for urban poor children during this period.

Despite significant residual disparities, there were noticeable pro-poor improvements in maternal education and maternal BMI during 2000-2018, which helped decrease the intra-urban HAZ gap. We speculate the changes in maternal BMI as an overall marker of improved household food access, which also affect child’s daily food intake and nutritional status. Other studies have remarked on significantly improved food security in Bangladesh during this time—largely resulting from rapid agricultural development and increased rice productivity—which broadly expanded food access in Bangladesh [39, 40]. The rising proportions of overweight mothers among both urban poor and urban non-poor, however, is a matter of growing public health concern, despite its positive association with child linear growth trends. Although obesity prevalence is higher among the urban non-poor, they have also increased rapidly among the urban poor. Whereas food programs expanding staple crops have improved maternal undernutrition, they may also have inadvertently increased obesity through promotion of cheaper but less varied and energy dense diets [13]. Given that stunted growth in children is a determinant of obesity in adulthood [2, 12, 41], the increased stunting risk among urban poor children may also heighten their susceptibility to developing obesity later in life. Thus, promotion of more optimal diets and obesity prevention need increased integration in food programs targeting the urban poor.

While the definitive pathways through which maternal education benefit child nutritional status remain unclear [42–44], we theorize that the rise in education and literacy among the urban poor mothers likely facilitated their access to relevant media and nutrition-promoting knowledge. For example, a slum-based study found no direct effect of maternal education on child nutrition after adjusting for maternal knowledge about child health [45]. Improved maternal education could have also led to better employment opportunities for mothers, therefore improving household income linked to nutritional improvements [38, 42]. However, this is unlikely to be the major pathway given prevailing cultural limitations and low female labor force participation in Bangladesh [46]. Furthermore, we found a positive association between maternal employment and lower levels of maternal education in this data, suggesting mothers with less education, and presumably poorer, are more likely to be working than those of higher socio-economic class.

Increased institutional births contributed to linear growth gains among urban poor children during 2000-2018, but the pace of progress in this area for the urban poor lagged significantly behind that of the non-poor and widened the intra-urban HAZ gap. In 2018, a large majority (67%) of births among the urban poor still took place at home. Child’s institutional birth—and corresponding link with antenatal care—is not only important for increasing chances of safe delivery, but often marks the first connection with the health system for the child that eases subsequent health service utilization, including nutritional counselling, vaccinations, and care for sickness and infections, which can all help downstream in improving child nutritional status. The increase in institutional deliveries among the urban poor was likely aided by the large-scale expansion of NGO-run health facilities that focused on maternal and child health and specifically targeted the urban poor [47, 48]. However, both demand- and supply-side barriers remain in adequate promotion of facility-based births among the urban poor with continually expanding urbanization [47, 49].

Evidence across countries and in Bangladesh points to increased household wealth—implying household’s ability to afford better commodities and services related to improved child nutrition—as a key driver of improved linear growth outcomes [37–39]. Our pooled data showed household wealth index as a key predictor of the intra-urban HAZ gap. However, our measurements of asset index and poverty cut-offs do not allow reliable interpretations about changes in urban wealth inequality during this period. Nevertheless, our analysis did not show a worsening urban wealth gap over time, which would have widened the intra-urban HAZ gap. Between 2000 and 2018, there was a decreased concentration of urban non-poor households in richest national wealth quintile (possibly due to previously poor households rising above poverty lines), alongside an increased concentration of urban poor households in the poorest national wealth quintile (potentially because of large growth in urban population and influxes of ‘new’ urban poor from rural areas). The net effect of these relative distributional changes in asset quintile helped reduce the intra-urban HAZ gap during this period. However, the rise in share of urban poor households in poorer wealth quintiles in our analysis decreased the average linear growth among urban poor children.

Our analysis of explanatory factors driving the linear growth gap between urban poor and non-poor children is generally comparable with a previous finding at the national level, which suggested household wealth, maternal education, maternal nutrition, and health service access as major contributors to socio-economic inequities in child linear growth status [23]. However, our finding of a reduced intra-urban child linear growth gap during 2000-2018 departs from the earlier national level findings of an increased difference in absolute stunting prevalence between poorest and richest wealth quintiles [23]. Yet, it is generally consistent with slum-based findings that saw a reduced—albeit marginally—intra-urban gap between slum- and non-slum children in mean HAZ during 2006-2013 [19, 50].

Our results also showed a significant improvement in average linear growth over time among urban poor children, consistent with trends at the national level as well as among slum children [19, 39]. Increased household wealth and maternal education were commonly the most important drivers explaining these linear growth improvements [19, 23, 28, 39, 40]. Progress in these factors were attributed to broad economic and social development [39, 40], including in slums, which saw improvements in living conditions over time [24, 29, 50]. In comparison, our findings attributed improved linear growth among urban poor children to primarily changes in maternal BMI, maternal education, and institutional deliveries, with a large residual of unexplained factors. Although our study did not review absolute changes in household wealth of urban poor, increased levels of maternal BMI could imply improvements in household food access stemming from general household economic progress and increased purchasing power. Taken as such, our findings among the urban poor are also consistent with existing global evidence on key drivers of national declines in stunting prevalence, which include improvements in household wealth, parental education, and access to reproductive health services [37].

### Limitations

Our study has several limitations: First, our asset-based definition of urban poor may underestimate the multi-dimensional aspects of urban poverty, including which is slum- or neighborhood-based. Compared to non-slum urban poor children, urban poor children in slums may be subject to increased spatial or residential risk of infection and undernutrition, which was not directly accounted for in this study. Second, the BDHS asset-based wealth index is distinct from HIES consumption-based measure of poverty. Therefore, applying the HIES poverty estimates directly to the BDHS sample may over- or under-estimate the quantification of the urban poor below official poverty lines, although there is comparability between asset- and expenditure-based indices in health inequality measurements [51]. Third, our empirical models were limited in terms of variable numbers and specifications, which may not reflect lived realities of urban poor, and we may thus miss or underestimate some important effects. Our focus on national-level predictors may also overlook variations by residence or poverty status in subgroup-level analyses. Additionally, we excluded community-level factors and interactions among variables that are also important in explaining variations [52]. Lastly, given its observational design, the associations shown in this study are subject to confounding bias and do not suggest causal interpretation.

## Conclusion

Bangladesh attained a significant net reduction in intra-urban child linear growth gap over the past 20 years. It seems to have achieved this indirectly and largely through pro-poor investments to improve broader determinants, such as girls’ education, maternal and child health, and household food security. Further improvements in these areas for the urban poor would aid in narrowing the remaining gap.

## Supplementary Information

**Additional file 1.** Supplementary material

## Data Availability

The datasets analyzed during the current study are available in the DHS Program repository, https://dhsprogram.com/.

## Abbreviations

BMI: body mass index
BDHS: Bangladesh Demographic and Health Survey
CI: confidence interval
COVID: corona virus disease
IYCF: Infant and Young Child Feeding
HAZ: height-for-age z-score
HIES: Household Income and Expenditure Survey
NGO: non-government organization
OLS: Ordinary Least Squares
OR: odds ratio
SD: standard deviation
SDG: Sustainable Development Goals
UNICEF: United Nations Children’s Fund
WHO: World Health Organization

## References

[1] United Nations. Transforming Our World: The 2030 Agenda for Sustainable Development. New York: UN Publishing; 2015.

[2] Black RE, Victora CG, Walker SP, Bhutta ZA, Christian P, de Onis M (2013). Maternal and child undernutrition and overweight in low-income and middle-income countries. Lancet.

[3] Black RE, Morris SS, Bryce J (2003). Where and why are 10 million children dying every year. Lancet.

[4] Stenberg K, Axelson H, Sheehan P, Anderson I, Gülmezoglu AM, Temmerman M (2014). Advancing social and economic development by investing in women's and children's health: a new Global Investment Framework. Lancet.

[5] Grantham-McGregor S, Cheung YB, Cueto S, Glewwe P, Richter L, Strupp B (2007). Developmental potential in the first 5 years for children in developing countries. Lancet.

[6] World Health Organization (WHO). WHO child growth standards and the identification of severe acute malnutrition in infants and children: a joint statement. Geneva: WHO; 2009. 24809116

[7] Victora CG, Adair L, Fall C, Hallal PC, Martorell R, Richter L (2008). Maternal and child undernutrition: consequences for adult health and human capital. Lancet.

[8] Crookston BT, Schott W, Cueto S, Dearden KA, Engle P, Georgiadis A (2013). Postinfancy growth, schooling, and cognitive achievement: Young Lives. Am J Clin Nutr.

[9] Fink G, Rockers PC (2014). Childhood growth, schooling, and cognitive development: further evidence from the Young Lives study. Am J Clin Nutr.

[10] United Nations Children’s Fund (UNICEF), World Health Organization (WHO), International Bank for Reconstruction and Development/The World Bank. Levels and trends in child malnutrition: Key Findings of the 2020 Edition of the Joint Child Malnutrition Estimates. Geneva: WHO; 2020.

[11] Development Initiatives. 2020 Global Nutrition Report: Action on equity to end malnutrition. Bristol: Development Initiatives; 2020.

[12] Victora CG, Christian P, Vidaletti LP, Gatica-Domínguez G, Menon P, Black RE. Revisiting maternal and child undernutrition in low-income and middle-income countries: variable progress towards an unfinished agenda. Lancet. 2021. 10.1016/S0140-6736(21)00394-9 PMC7613170 33691094

[13] Perez-Escamilla R, Bermudez O, Buccini GS, Kumanyika S, Lutter CK, Monsivais P (2018). Nutrition disparities and the global burden of malnutrition. BMJ.

[14] Ernst KC, Phillips BS, Duncan BD (2013). Slums are not places for children to live: vulnerabilities, health outcomes, and possible interventions. Adv Pediatr.

[15] Ezeh A, Oyebode O, Satterthwaite D, Chen Y-F, Ndugwa R, Sartori J (2017). The history, geography, and sociology of slums and the health problems of people who live in slums. Lancet.

[16] Lilford RJ, Oyebode O, Satterthwaite D, Melendez-Torres GJ, Chen Y-F, Mberu B (2017). Improving the health and welfare of people who live in slums. Lancet.

[17] Mberu BU, Haregu TN, Kyobutungi C, Ezeh AC (2016). Health and health-related indicators in slum, rural, and urban communities: a comparative analysis. Glob Health Action..

[18] Agarwal S (2011). The state of urban health in India; comparing the poorest quartile to the rest of the urban population in selected states and cities. Environ Urban.

[19] Raju D, Kim KY, Nguyen Q, Govindaraj R. Cities, slums, and early child growth: empirical evidence from Bangladesh. World Bank Policy Research Working Paper [Internet]. 2017 Jun 8(8094) [cited 2020 August 2020]. Available from: https://openknowledge.worldbank.org/handle/10986/27294.

[20] NIPORT, Mitra and Associates, and ICF International. Bangladesh Demographic and Health Survey 2000. Dhaka, Bangladesh, and Rockville, Maryland, USA; 2000.

[21] NIPORT, Mitra and Associates, and ICF International. Bangladesh Demographic and Health Survey 2017-2018. Dhaka, Bangladesh, and Rockville, Maryland, USA; 2020.

[22] United Nations Children’s Fund, World Health Organization, and World Bank. Joint child malnutrition estimates (country level) [dataset]. 2020 July [cited 2020 December 15]. United Nations Children’s Fund. Available from: https://data.unicef.org/resources/dataset/malnutrition-data/.

[23] Rabbani A, Khan A, Yusuf S, Adams A (2016). Trends and determinants of inequities in childhood stunting in Bangladesh from 1996/7 to 2014. Int J Equity Health..

[24] National Institute of Population Research and Training (NIPORT), International Centre for Diarrhoeal Disease Research, Bangladesh (ICDDR,B), and MEASURE Evaluation. Bangladesh Urban Health Survey 2013 Final Report. Dhaka, Bangladesh and Chapel Hill, North Carolina, USA: NIPORT, icddr,b, and MEASURE Evaluation; 2015.

[25] Wratten E (1995). Conceptualizing urban poverty. Environ Urban.

[26] World Health Organization (WHO) and United Nations Human Settlements Programme (UN-HABITAT). Hidden Cities: Unmasking and Overcoming Health Inequities in Urban Settings. Geneva: WHO and UN-HABITAT; 2010.

[27] World Health Organization (WHO) and United Nations Human Settlements Programme (UN-HABITAT). Global Report on Urban Health: Equitable, Healthier Cities for Sustainable Development. Geneva: WHO; 2016.

[28] Ahsan KZ, Arifeen SE, Al-Mamun MA, Khan SH, Chakraborty N (2017). Effects of individual, household and community characteristics on child nutritional status in the slums of urban Bangladesh. Arch Public Health..

[29] National Institute of Population Research and Training (NIPORT), MEASURE Evaluation, International Centre for Diarrhoeal Disease Research, Bangladesh (ICDDR,B), and Associates for Community and Population Research (ACPR). 2006 Bangladesh Urban Health Survey. Dhaka, Bangladesh and Chapel Hill, North Carolina, USA: NIPORT, MEASURE Evaluation, ICDDR,B, and ACPR; 2008.

[30] Fink G, Gunther I, Hill K (2014). Slum residence and child health in developing countries. Demography.

[31] Nolan L (2015). Slum Definitions in Urban India: Implications for the Measurement of Health Inequalities. Popul Dev Rev.

[32] Government of Bangladesh (GOB). Final report on Household Income and Expenditure Survey 2016. Dhaka: Bangladesh Bureau of Statistics, Ministry of Planning, GOB; 2019.

[33] Black RE, Allen LH, Bhutta ZA, Caulfield LE, de Onis M, Ezzati M (2008). Maternal and child undernutrition: global and regional exposures and health consequences. Lancet.

[34] United Nations Children’s Fund (UNICEF). Strategy for improved nutrition of children and women in developing countries. New York: UNICEF; 1990. 10.1007/BF02810402 1937618

[35] StataCorp (2017). Stata Statistical Software: Release 15.

[36] Uwiringiyimana V, Ocke MC, Amer S, Veldkamp A (2019). Predictors of stunting with particular focus on complementary feeding practices: A cross-sectional study in the northern province of Rwanda. Nutrition.

[37] Vaivada T, Akseer N, Akseer S, Somaskandan A, Stefopulos M, Bhutta ZA (2020). Stunting in childhood: an overview of global burden, trends, determinants, and drivers of decline. Am J Clin Nutr.

[38] Headey D, Hoddinott J, Park S (2017). Accounting for nutritional changes in six success stories: A regression-decomposition approach. Glob Food Secur.

[39] Headey D, Hoddinott J, Ali D, Tesfaye R, Dereje M (2015). The Other Asian Enigma: Explaining the Rapid Reduction of Undernutrition in Bangladesh. World Dev.

[40] Nisbett N, Davis P, Yosef S, Akhtar N (2017). Bangladesh’s story of change in nutrition: Strong improvements in basic and underlying determinants with an unfinished agenda for direct community level support. Glob Food Secur.

[41] Martins VJ, Toledo Florencio TM, Grillo LP, PFM d C, Martins PA, Clemente AP (2011). Long-lasting effects of undernutrition. Int J Environ Res Public Health.

[42] Ruel MT, Alderman H (2013). Nutrition-sensitive interventions and programmes: how can they help to accelerate progress in improving maternal and child nutrition. Lancet.

[43] Headey DD (2013). Developmental Drivers of Nutritional Change: A Cross-Country Analysis. World Dev.

[44] Webb P, Block SA (2004). Nutrition information and formal schooling as inputs to child nutrition. Econ Dev Cult Change.

[45] Fakir AM, Khan MW (2015). Determinants of malnutrition among urban slum children in Bangladesh. Health Econ Rev.

[46] Kotikula A, Hill R, Raza WA (2019). What Works for Working Women: Understanding Female Labor Force Participation in Urban Bangladesh.

[47] Adams AM, Islam R, Ahmed T (2015). Who serves the urban poor? A geospatial and descriptive analysis of health services in slum settlements in Dhaka, Bangladesh. Health Policy Plan.

[48] Albis MLF, Bhadra SK, Chin B (2019). Impact evaluation of contracting primary health care services in urban Bangladesh. BMC Health Serv Res.

[49] Asian Development Bank (ADB). Report and recommendation of the president to the Board of Directors on a proposed loan to the People’s Republic of Bangladesh for the additional financing of the Urban Primary Health Care Services Delivery Project. Manila: ADB; 2018.

[50] Angeles G, Ahsan KZ, Streatfield PK, El Arifeen S, Jamil K (2019). Reducing Inequity in Urban Health: Have the Intra-urban Differentials in Reproductive Health Service Utilization and Child Nutritional Outcome Narrowed in Bangladesh. J Urban Health.

[51] Wagstaff AVDE, Watanabe N (2003). On decomposing the causes of health sector inequalities with an application to malnutrition inequalities in Vietnam. J Econom.

[52] Diez-Roux AV (1998). Bringing Context Back into Epidemiology: Variables and Fallacies in Multilevel Analysis. Am J Public Health.

