# Supplementary material for: Understanding urban inequalities in children’s linear growth outcomes: a trend and decomposition analysis of 39,049 children in Bangladesh (2000-2018)
Source: BMC Public Health. 2021 Nov 30;21:2192. doi: 10.1186/s12889-021-12181-x (PMC8631262; doi:10.1186/s12889-021-12181-x)
Supplement: Supplementary file 1 — Additional file 1. Supplementary material [file 12889_2021_12181_MOESM1_ESM.docx]

**Supplementary Material**

**Additional file 1: Appendix**

| Appendix 1: Percentage urban population below the ‘upper’ poverty line in HIES 2000 to 2018 and the corresponding asset score cutoff values by each BDHS survey year | | | | | |
| --- | --- | --- | --- | --- | --- |
| DHS survey year | Corresponding HIES survey year | HIES, Urban, Head Count Rate of poverty incidence (upper poverty line, CBN Method),% | DHS asset score cutoff value | DHS Urban mean Asset Score  (national mean asset score) | DHS Urban Poor Mean Asset Score (average asset index) |
| DHS 1999-2000 | HIES 2000 | 35.2 | 13707 | 83469 (-15627) | -47417 (2.55) |
| DHS 2004 | HIES 2005 | 28.4 | -27923 | 71427 (-13597) | -68276 (1.91) |
| DHS 2007 | HIES 2005 | 28.4 | -25484 | 79751 (-15712) | -57286 (2.46) |
| DHS 2011 | HIES 2010 | 21.3 | -16141 | 85486 (-8144) | -60943 (2.17) |
| DHS 2014 | HIES 2016 | 18.9 | -29154 | 80739 (-2624) | -76060 (1.92) |
| DHS 2018 | HIES 2016 | 18.9 | -35746 | 72999 (-3579) | -79203 (1.88) |

| Appendix 2: Details of Variable Specifications | |
| --- | --- |
| *Variables* | *Outcome* |
| Stunting | Height-for-age z score (HAZ) of more than two standard deviations (SD) below international reference median (*2006 WHO Child Growth Standards).* The BDHS collects anthropometric data on under-five children and converts the measurements into standard indices of height-for-age, weight-for-height, and weight-for-age based on 2006 WHO reference population. For BDHS survey rounds conducted prior to the promulgation of the *2006 WHO Child Growth Standards*, the WHO z-scores are retrospectively calculated by the DHS Program*.* |
| HAZ | Height-for-age *z* scores (HAZ), according to 2006 WHO growth standards |
| *Household level* | |
| national wealth quintiles | The wealth quintiles indicate relative household wealth at the national level, ordered from 1 (poorest) to 5 (richest) in BDHS. |
| household size | Household size is a quantitative variable in BDHS indicating number of household members, which we recoded as either average or below (1 to 6 members) or above average size (7 or more members). (Average household size is 6.4 in pooled data.) |
| household head | Household head indicates either a male or a female head of household. |
| administrative division | Administrative divisions of Bangladesh. There were 6 administrative divisions (Dhaka, Barisal, Chittagong, Khulna, Rajshahi and Sylhet) in DHS surveys 2000, 2004, and 2007. DHS surveys 2011 and 2014 included 7 administrative divisions (adding Rangpur Division), and DHS survey 2018 included 8 divisions (adding Mymensingh Division). |
| drinking water facilities | Drinking water facilities include two variables on source of drinking water, and location of the water source (whether piped into own house, yard, or plot, or located elsewhere).  ‘Improved’ sources of drinking water include piped water (public or private), tube well or borehole, protected well or spring, rainwater, and bottled water. Non-improved sources are unprotected well, tanker truck or cart with drum, and surface water. |
| Toilet facilities | Toilet facilities include two variables on whether the toilet is an ‘improved’ or a ‘non-improved’ type, and whether it is shared or not shared with other households.  Improved’ toilet types include flush toilet piped to a sewer system, septic tank, or latrine, ventilated improved pit latrine, pit latrine with a slab, and composting toilet. ‘Non-improved’ toilet types were flush toilet to ‘somewhere else’ or ‘don’t know where’, pit latrine without slab or open pit, and hanging toilet or latrine. Households relying on open defecation (‘no facility/bush/field’) were also included as having non-improved types. |
| Place of handwashing | Place of handwashing is included as an additional hygiene element, and considered as ‘in dwelling’ if it was observed by the interviewer inside the house, and ‘not in dwelling’ if it was not observed by the interviewer in the house (observations for which handwashing place was not observed for reasons other than ‘not in dwelling’ were excluded from analysis). |
| Cooking facilities | Cooking facilities include two separate variables on the type of cooking fuel used in household (recoded as either solid or non-solid based on standard definition) and the place of cooking (indicating whether food is usually cooked in the house, in separate building, or outdoors). |
| *Individual mother’s* level | |
| maternal education | Maternal education indicates the highest education level attended. The variable is standardized into following categories in DHS: No education, Primary, Secondary, and Higher. The categories were compared after the regression model and further combined into binary terms of ‘no or primary level education’ and ‘secondary or higher-level education’ in the final analysis. |
| media exposure | Media exposure is an aggregated variable derived from separate variables in BDHS capturing frequency of reading newspaper or magazine, watching television, or listening to radio. A mother has ‘some exposure’ to media if she reports reading, listening, or watching the media at some regular interval (less than weekly, at least once weekly, or almost every day). Mothers with ‘some exposure’ are likely to have some access to media, whereas mothers reporting ‘not at all’ are unlikely to have access to media. |
| age at first marriage  age at first child’s birth | Mother’s age at first marriage and age at first child’s birth are quantitative variables in BDHS, which we recoded into binary terms of before (17 or younger), or after (18 or older) reaching the legal age of adulthood. Consistent with studies showing link between adolescent pregnancies and child stunting, non-parametric estimates of the quantitative age variables in BDHS depict a non-linear relationship with stunting (showing higher risk of child stunting for mothers marrying or having first children while under-18 years than when 18 years or older, but the risk inclines again after reaching the age of 30.) (We did not exclude the more advanced maternal ages as these were only a small number of observations.) |
| children ever born | Total children ever born to mothers are continuous variables in BDHS, showing positive linear relationship with stunting in non-parametric estimates, and we separated the values into three quantiles. |
| children living at home | Total number of children living at home with mother (not just under-5 children) is included as a more direct indicator of total child dependency burden; the variable is derived from aggregating BDHS quantitative variables of total sons and total daughters at home. |
| contraception use | Current contraception use indicates whether or not the mother was using any contraceptive method, including natural methods, at the time of interview; we included this variable as a proximate indicator of family planning use and mother’s control over own reproductive circumstances. |
| employment status | We included maternal employment given higher levels of mothers working seems to be a peculiar feature of urban poverty. A mother is considered to have positive employment status if she reported working any amount (currently, in past year, have job but on leave) and in any type of income generating occupation in the last 12 months of interview. |
| level of autonomy | Mother’s level of autonomy is an aggregated scale based on variables collected in BDHS on mother’s decision-power related to own healthcare, large household purchases, and visits to family and relatives (equal value given to each item). ‘High’ power indicates respondent mother alone can make decision; ‘average’ power indicates jointly with husband/partner; ‘low’ power indicates husband alone or someone else make the decisions*.* |
| nutritional status  (BMI and stature) | Maternal height (stature) is a continuous variable in BDHS, which we recoded as categorical variables of ‘very short’ (<145 cm), ‘short’ (145 to <155 cm), or normal to tall (155 to 200 cm). We used the reference of ≥155 cm as normal height, although the average height of Bangladeshi women are in the ‘short’ category.  BMI is given in BDHS as a quantitative variable derived from maternal weight divided by height, and we recoded this into three categories of underweight (<18.5), normal (18.5 to <25), and overweight (≥25) according WHO references (pregnant women are excluded). |
| maternal health seeking | We included number of antenatal care visits (for last birth) as an indicator of maternal health seeking, which we recoded a ‘none’, ‘1-3’, or ‘4 or more’ visits, the latter being the WHO standard recommendation on number of contacts. |
| *Individual child’s* level | |
| age | Child’s age is a quantitative variable in BDHS, which we recoded into three age groups of 0-11 and 12-23 months—when children are most vulnerable to stunting—and a broader 24-59 months. |
| order of birth | Child’s birth order was recoded into binary groups of ‘1 to 2’ or ‘3 or more’, which roughly represents above or below the average birth order in pooled data. |
| place of birth | Child’s place of birth is recoded in binary terms as either at home or at a facility (including public, private, and NGO facilities from primary to tertiary levels); we excluded ‘other.’ NGO supported ‘birthing huts’ were counted as home births. |
| perceived size at birth | Child’s size at birth is perceived and reported by mother and recoded into three categories of very small/smaller than average, average, and very large or larger than average. |
| vaccination status | A child is considered to have received measles vaccination at any time before survey if the vaccination is marked or dated on card or reported by mother. We selected measles as a proxy for full vaccination as it is the last vaccine of all basic vaccines given to children typically before 12 months. As the vaccine was typically given after 9 months of age, children 8 months or younger were counted as having received measles vaccination. Government considers ‘fully vaccinated’ if all basic vaccines (BCG; 3 doses each of pentavalent and polio; measles) have been administered. |
| deworming status | A child is considered to have had drugs for intestinal parasites in last 6 months if reported by mother. |

| **Appendix 3**: (Unadjusted Associations) *Stunting-Crude Odds Ratio (95% CI)* | | | |
| --- | --- | --- | --- |
| *N (unweighted)* | *39049* | *12198* | *26851* |
| *Predictors* | *National* | *Urban Residence* | *Rural Residence* |
| *Child’s Background* | | | |
| **Sex** Male  Female | 1  0.97 (0.93,1.01) | 1  0.95 (0.88,1.02) | 1  0.98 (0.93,1.03) |
| **Age (months)**  0-11  12-23  24-59 | 1  2.84***(2.64,3.06)  3.46***(3.12,2.92) | 1  2.72***(2.37,3.13)  2.70***(2.39,3.05) | 1  2.92***(2.68,3.19)  3.35***(3.10,3.61) |
| **Birth Order** 1 to 2  3 or higher | 1  1.49***(1.43,1.56) | 1  1.63***(1.50,1.78) | 1  1.39***(1.32,1.46) |
| **Place of birth** Health Facility  Home | 1  2.47***(2.33,2.62) | 1  2.62***(2.38,2.88) | 1  2.18***(2.02,2.36) |
| **Vaccination (measles)** Yes  No | 1  1.66***(1.55,1.78) | 1  1.90***(1.68,2.16) | 1  1.54***(1.42,1.67) |

| *Mother’s Background* | | | |
| --- | --- | --- | --- |
| **Age at first marriage**  18 years or older  17 years or younger | 1  1.57***(1.49,1.66) | 1  1.89***(1.71,2.08) | 1  1.35***(1.26,1.44) |
| **Age at first child’s birth**  18 years or older  17 years or younger | 1  1.45***(1.39,1.52) | 1  1.71***(1.58,1.85) | 1  1.31***(1.25,1.38) |
| **Highest education level**  Secondary or higher  No education/ Primary only | 1  2.33***(2.22,2.44) | 1  2.78***(2.55,3.02) | 1  2.08***(1.96,2.20) |
| **Media exposure** Some  Not at all | 1  1.73***(1.65,0.81) | 1  1.81***(1.63,2.02) | 1  1.58***(1.50,1.66) |
| **Marital status** Married  Widowed/divorced/separated | 1  1.31**(1.11,1.54) | 1  1.70***(1.28,2.25) | 1  1.17 (0.96,1.42) |
| **Total no. children living with mother**  3 or less  4 or more | 1  1.85***(1.75,1.97) | 1  2.17***(1.91,2.47) | 1  1.69***(1.59,1.81) |
| **Total children ever born** 1 to 2  3  4 or more | 1  1.24***(1.17,1.31)  1.93***(1.83,2.03) | 1  1.20***(1.08,1.34)  2.26***(2.03,2.52) | 1  1.22***(1.14,1.31)  1.74***(1.64,1.86) |
| **Current contraception use**  Not currently using  Currently using (any method) | 1  0.98 (0.93,1.02) | 1  1.00 (0.91,1.09) | 1  1.02 (0.97,1.08) |
| **Maternal stature**  Normal/Tall (155 to <200 cm)  Short (145 to <155 cm)  Very short (<145 cm) | 1  2.21***(2.09,2.34)  4.99***(4.62,5.39) | 1  2.38***(2.13,2.65)  5.47***(2.13,6.65) | 1  2.14***(2.00,2.29)  4.81***(4.38,5.28) |
| **Body Mass Index**  Normal (18.5 to <25)  Underweight (<18.5)  Overweight (≥25) | 1  1.58***(1.50,1.66)  0.48***(0.45,0.52) | 1  1.80***(1.63,1.99)  0.49***(0.43,0.54) | 1  1.45***(1.37,1.53)  0.51***(0.46,0.56) |
| **Worked in last 12 months** No  Yes | 1  1.04 (0.99,1.09) | 1  1.26***(1.14,1.39) | 1  0.95 (0.90,1.02) |
| **Level of autonomy** Low  Average-high | 1  0.84***(0.80,0.88) | 1  0.79***(0.73,0.87) | 1  0.90***(0.85,0.94) |

| *Household Background* | | | |
| --- | --- | --- | --- |
| **National Wealth Index** Poor  Poorer  Middle  Richer  Richest | 1  0.78***(0.73,0.84)  0.62***(0.58,0.67)  0.49***(0.46,0.53)  0.28***(0.26,0.30) | 1  0.86*(0.72,1.02)  0.66***(0.56,0.78)  0.56***(0.47,0.65)  0.29***(0.24,0.33) | 1  0.77***(0.72,0.83)  0.62***(0.57,0.67)  0.47***(0.43,0.51)  0.28***(0.25,0.31) |
| **Residence** Urban  Rural | 1  1.46***(1.36,1.55) | - | - |
| **Division** Dhaka  Chittagong  Others | 1  1.07(0.97,1.18)  0.95(0.88,1.03) | 1  1.19^(1.00,1.42)  0.94(0.82,1.08) | 1  0.97(0.86,1.10)  0.89*(0.81,0.99) |
| **Household size**  1 to 6  7 or more | 1  1.03 (0.99,1.08) | 1  0.99 (0.90,1.07) | 1  1.03 (0.98,1.09) |
| **Household head** Male  Female | 1  0.81***(0.75,0.87) | 1  0.84** (0.73,0.96) | 1  0.79***(0.72,0.86) |
| **Type of toilet facility** Improved  Non-improved | 1  1.93***(1.84,2.03) | 1  1.81***(1.64,2.00) | 1  1.86***(1.75,1.97) |
| **Source of drinking water** Improved  Non-improved | 1  1.55***(1.33,1.80) | 1  1.25 (0.92,1.70) | 1  1.48***(1.26,1.74) |

Notes: Observations are live children with valid anthropometirc data. Standard errors clustered at the survey-cluster level. ^*p* ≤0.1; **p* ≤0.05; ** *p* ≤0.01; *** *p* ≤0.001

| **Appendix 4:** Adjusted estimates with all covariates (Stepwise model selection) | | |  |
| --- | --- | --- | --- |
|  | *National* | *Urban Residence* | *Rural Residence* |
| *N (unweighted)* | 30463 | 9425 | 21038 |
| *Predictors* | *Stunting OR (95% CI)* | | |
| Child Background | | | |
| **Sex** Male Female | 1:00  0.93**(0.88,0.97) | 1:00  0.89**(0.81,0.98) | - |
| **Age (months)** 0-11  12-23  24-59 | 1:00  3.25***(2.99,3.53)  3.77***(3.49,4.06) | 1:00  3.18***(2.72,3.73)  3.24***(2.81,3.73) | 1:00  3.28***(2.98,3.62)  4.02***(3.68,4.39) |
| **Place of birth** Health Facility  Home | 1:00  1.27***(1.18,1.37) | 1:00  1.37***(1.22,1.55) | 1:00  1.17***(1.07,1.29) |
| **Vaccinated (measles)** Yes  No | 1:00  1.22***(1.13,1.32) | 1:00  1.34***(1.16,1.54) | 1:00  1.18***(1.08,1.29) |
| Mother’s Background | | | |
| **Age at first marriage**  18 years or older  17 years or younger | - | 1:00  1.23***(1.09,1.39) | - |
| **Age at first child’s birth**  18 years or older  17 years or younger | 1:00  1.06*(1.00,1.12) | - | - |
| **Highest education level**  Secondary or higher  No education/ Primary only | 1:00  1.22***(1.14,1.30) | 1:00  1.34***(1.20,1.50) | 1:00  1.13**(1.05,1.22) |
| **Media exposure** Some  Not at all | 1:00  1.08**(1.02,1.14) | - | 1:00  1.13***(1.06,1.21) |
| **No. children living with mother**  3 or less  4 or more | 1:00  1.18***(1.10,1.27) | - | 1:00  1.13**(1.04,1.23) |
| **Total children ever born** 1 to 2  3  4 or more | - | 1:00  -  1.33***(1.18,1.50) | - |
| **Maternal stature**  Normal/Tall (155 to <200 cm)  Short (145 to <155 cm)  Very short (<145 cm) | 1:00  2.14***(2.00,2.28)  4.71***(4.30,5.16) | 1:00  2.30***(2.03,2.61)  5.35***(4.54,6.30) | 1:00  2.07***(1.92,2.24)  4.45***(3.99,4.96) |
| **Body Mass Index**  Normal (18.5 to <25)  Underweight (<18.5)  Overweight (≥25) | 1:00  1.30***(1.23,1.38)  0.71***(0.65,0.78) | 1:00  1.47***(1.31,1.65)  0.74***(0.64,0.85) | 1:00  1.25***(1.17,1.34)  0.70***(0.62,0.80) |
| **Level of autonomy** Low  Average-high | 1:00  0.94*(0.89,0.99) | 1:00  0.90*(0.81,0.99) | - |
| Household Background | | | |
| **Wealth Index (national)** Poor  Poorer  Middle  Richer  Richest | 1:00  0.87***(0.80,0.94)  0.79***(0.73,0.86)  0.69***(0.63,0.76)  0.48***(0.43,0.54) | 1:00  -  0.79**(0.68,0.93)  0.75***(0.65,0.87)  0.52***(0.45,0.61) | 1:00  0.86***(0.79,0.93)  0.78***(0.71,0.85)  0.63***(0.57,0.71)  0.44***(0.37,0.51) |
| **Household size**  1-6  7 or more | 1:00  1.07* (1.01,1.14) | - | 1:00  1.12***(1.05,1.21) |
| **Residence** Urban  Rural | - | - | - |
| **Division** Dhaka  Chittagong  Others | 1:00  -  0.87***(0.82,0.92) | 1:00  -  0.79***(0.71,0.89) | 1:00  1.16***(1.06,1.26)  - |
| **Source of drinking water** Improved  Non-improved | 1:00  1.24**(1.06,1.44) | - | 1:00  1.31**(1.11,1.54) |
| **Type of toilet facility** Improved  Non-improved | 1:00  1.12***(1.05,1.19) | - | 1:00  1.15***(1.06,1.23) |

Notes: Observations are live children with valid anthropometirc data. Model accounts for survey fixed effect and standard errors clustered at the survey-cluster level. Estimates of variables that did not survive the stepwise model selection are not presented in the table. **p* ≤0.05; ** *p* ≤0.01; *** *p* ≤0.001

| **Appendix 5:** Estimated contributions of national-level stunting predictors to urban poor and non-poor stunting gap, pooled urban sample (2000-2018) | | | | | | | | | | | | |
| --- | --- | --- | --- | --- | --- | --- | --- | --- | --- | --- | --- | --- |
| *Key stunting predictors*  **Urban poverty**  (urban poor)  **Child’s sex**  (Female)  **Child’s age**  (12-23 months)  (24-59 months)  **Health service use** Child’s place of delivery (at home)  Child’s vaccination  (not vaccinated)  **Maternal education**  (no/primary)  **Maternal nutrition**  BMI:  (underweight)  (overweight)  Stature:  (short: 145 to <155cm)  (very short: <145 cm)  **Mat. reproductive background**  Age at first child’s birth (≤17 years)  No. children living with mother (≥4)  **Mat. autonomy & media exposure**  Media exposure  (not at all)  Autonomy level  (average-high)  **Household WASH**  Toilet type  (non-improved)  Drinking water source (non-improved)  **Household Division**  Chittagong  Others  **Household SES**  Household size  (≥7 members)  Household wealth  (Poorer)  (Middle)  (Richer)  (Richest) | 1  (N=12198) | 2  (N=12198) | 3  (N=10252) | 4  (N=10233) | 5  (N=10231) | 6  (N=10217) | 7  (N=10201) | 8  (N=10194) | 9  (N=10099) | 10  (N=9426) | 11  (N=9426) | 12  (N=9426) |
|  | Odds Ratios (95% CI) | | | | | | | | | | | |
|  | 2.35***  (2.13,2.59)  --  --  --  --  --  --  --  --  --  --  --  --  --  --  --  -- | 2.45***  (2.22,2.71)  0.94^  (0.87,1.02)  2.90***  (2.52,3.34)  2.87***  (2.53,3.26)  --  --  --  --  --  --  --  --  --  --  --  --  --  -- | 2.01***  (1.81,2.24)  0.89**  (0.82,0.97)  2.98***  (2.58,3.44)  2.92***  (2.56,3.33)  2.00***  (1.81,2.22)  --  --  --  --  --  --  --  --  --  --  --  --  -- | 1.98***  (1.77,2.20)  0.88**  (0.81,0.96)  2.97***  (2.57,3.44)  2.93***  (2.57,3.35)  1.95***  (1.76,2.16)  1.56***  (1.36,1.79)  --  --  --  --  --  --  --  --  --  --  --  -- | 1.69***  (1.51,1.89)  0.89**  (0.82,0.97)  3.00***  (2.59,3.47)  2.93***  (2.56,3.35)  1.64***  (1.48,1.83)  1.43***  (1.25,1.63)  1.82***  (1.65,2.02)  --  --  --  --  --  --  --  --  --  --  -- | 1.56***  (1.40,1.74)  0.89**  (0.82,0.97)  2.98***  (2.58,3.46)  3.04***  (2.66,3.48)  1.51***  (1.36,1.68)  1.39***  (1.21,1.59)  1.75***  (1.58,1.94)  1.43***  (1.29,1.60)  0.69***  (0.61,0.79)  --  --  --  --  --  --  --  --  --  -- | 1.53***  (1.37,1.72)  0.89**  (0.82,0.97)  3.15***  (2.71,3.66)  3.27***  (2.85,3.75)  1.52***  (1.36,1.69)  1.37***  (1.19,1.57)  1.55***  (1.40,1.72)  1.52***  (1.36,1.70)  0.70***  (0.62,0.80)  2.33***  (2.07,2.63)  5.26***  (4.49,6.16)  --  --  --  --  --  --  --  --  -- | 1.52***  (1.35,1.70)  0.89**  (0.82,0.98)  3.14***  (2.70,3.65)  3.24***  (2.82,3.71)  1.47***  (1.31,1.64)  1.36***  (1.18,1.56)  1.46***  (1.32,1.63)  1.51***  (1.35,1.69)  0.70***  (0.61,0.80)  2.35***  (2.08,2.65)  5.33***  (4.55,6.25)  1.11*  (1.01,1.23)  1.36***  (1.17,1.57)  --  --  --  --  --  --  -- | 1.52***  (1.35,1.72)  0.89**  (0.81,0.97)  3.13***  (2.69,3.65)  3.26***  (2.84,3.74)  1.47***  (1.32,1.65)  1.35***  (1.18,1.56)  1.48***  (1.33,1.65)  1.50***  (1.34,1.68)  0.70***  (0.62,0.81)  2.35***  (2.08,2.65)  5.31***  (4.53,6.23)  1.10^  (1.00,1.21)  1.34***  (1.15,1.55)  0.98  (0.85,1.11)  0.90*  (0.81,0.99)  --  --  --  --  -- | 1.47***  (1.29,1.67)  0.89*  (0.81,0.98)  3.14***  (2.69,3.68)  3.23***  (2.81,3.73)  1.48***  (1.32,1.67)  1.38***  (1.19,1.59)  1.45***  (1.30,1.63)  1.48***  (1.33,1.66)  0.72***  (0.62,0.82)  2.33***  (2.05,2.65)  5.39***  (4.57,6.36)  1.09^  (0.99,1.21)  1.35***  (1.16,1.56)  0.99  (0.87,1.14)  0.90*  (0.81,1.00)  1.11^  (0.99,1.24)  0.81  (0.55,1.20)  --  --  -- | 1.54***  (1.35,1.75)  0.89*  (0.81,0.98)  3.15***  (2.69,3.69)  3.25***  (2.82,3.75)  1.46***  (1.29,1.65)  1.36***  (1.18,1.57)  1.44***  (1.28,1.61)  1.50***  (1.34,1.68)  0.71***  (0.62,0.82)  2.32***  (2.04,2.64)  5.39***  (4.57,6.36)  1.10^  (1.00,1.22)  1.34***  (1.16,1.56)  1.00  (0.88,1.15)  0.90*  (0.81,0.99)  1.07  (0.96,1.20)  0.81  (0.55,1.20)  0.98  (0.83,1.17)  0.81**  (0.71,0.92)  --  -- | 1.33*  (1.03,1.72)  0.89*  (0.81,0.98)  3.17***  (2.71,3.71)  3.26***  (2.83,3.76)  1.40***  (1.24,1.58)  1.34***  (1.16,1.55)  1.38***  (1.23,1.55)  1.47***  (1.32,1.65)  0.74***  (0.64,0.85)  2.32***  (2.05,2.64)  5.40***  (4.57,6.37)  1.08^  (0.98,1.20)  1.34***  (1.16,1.56)  0.96  (0.84,1.10)  0.90^  (0.81,1.00)  1.03  (0.92,1.15)  0.81  (0.56,1.18)  0.95  (0.79,1.13)  0.77***  (0.67,0.88)  1.02  (0.91,1.14)  0.87 (0.71,1.07)  0.78*(0.64,0.96)  0.89 (0.66,1.20)  0.63**(0.45,0.86) |
| Predicted stunting gap (95% CI) | 2.35***  (2.13,2.59) | 2.45***  (2.22,2.71) | 2.01***  (1.81,2.24) | 1.98***  (1.77,2.20) | 1.69***  (1.51,1.89) | 1.56***  (1.40,1.74) | 1.53***  (1.37,1.72) | 1.52***  (1.35,1.70) | 1.52***  (1.35,1.72) | 1.47***  (1.29,1.67) | 1.54***  (1.35,1.75) | 1.33*  (1.03,1.72) |
| Additional variables’ contribution to stunting gap (%) | 100% | +7.4% | -32.6% | -2.2% | -21.5% | -9.6% | -2.2% | -1.0% | 0.0% | -3.7% | +3.0% | -15.6% |
| Total remaining gap |  | 107.4% | 74.8% | 72.6% | 51.1% | 41.5% | 39.3% | 38.3% | 38.3% | 34.6% | 37.6% | 22.0% |

Notes: HAZ (height-for-age z-scores according to WHO). Model include survey year fixed effects and standard errors clustered at the survey-cluster level. ^*p* ≤0.1; **p* ≤0.05; ** *p* ≤0.01; *** *p* ≤0.001. Columns show incremental adjustments for key stunting determinants identified from stepwise selection with 5% significance and resulting changes in stunting gap (odds ratio).

| Appendix 6: Predicted urban poor and non-poor stunting and linear growth gap (2000-2018) | | | |
| --- | --- | --- | --- |
| Year | Stunting Odds Ratio  (robust SE) | Linear Growth  HAZ OLS (robust SE) | N (not weighted) |
| 2000 | 3.09*** (0.38) | -0.79***(0.08) | 1366 |
| 2004 | 2.31***(0.30) | -0.71***(0.09) | 1771 |
| 2007 | 2.21***(0.29) | -0.60***(0.08) | 1850 |
| 2011 | 2.60***(0.29) | -0.75***(0.08) | 2342 |
| 2014 | 1.97***(0.22) | -0.45***(0.07) | 2188 |
| 2018 | 2.16***(0.28) | -0.52***(0.08) | 2681 |
| Pooled (2000-2018) | 2.35***(0.12) | -0.63***(0.03) | 12,274 |

^*p* ≤0.1; **p* ≤0.05; ** *p* ≤0.01; *** *p* ≤0.001; cluster adjusted at survey level

**Appendix 7: Correlation Matrix of Study Variables (Pairwise Correlations)**

| Variables | (1) | (2) | (3) | (4) | (5) | (6) | (7) | (8) | (9) | (10) | (11) | (12) | (13) | (14) | (15) | (16) | (17) | (18) | (19) | (20) | (21) | (22) | (23) | (24) | (25) | (26) |
| --- | --- | --- | --- | --- | --- | --- | --- | --- | --- | --- | --- | --- | --- | --- | --- | --- | --- | --- | --- | --- | --- | --- | --- | --- | --- | --- |
| (1) stunted | 1.00 |  |  |  |  |  |  |  |  |  |  |  |  |  |  |  |  |  |  |  |  |  |  |  |  |  |
| (2) child’s sex | -0.01 | 1.00 |  |  |  |  |  |  |  |  |  |  |  |  |  |  |  |  |  |  |  |  |  |  |  |  |
| (3) child’s age | 0.18* | 0.00 | 1.00 |  |  |  |  |  |  |  |  |  |  |  |  |  |  |  |  |  |  |  |  |  |  |  |
| (4) birth order | 0.10* | 0.00 | 0.03* | 1.00 |  |  |  |  |  |  |  |  |  |  |  |  |  |  |  |  |  |  |  |  |  |  |
| (5) delivery place | 0.18* | 0.02* | 0.12* | 0.21* | 1.00 |  |  |  |  |  |  |  |  |  |  |  |  |  |  |  |  |  |  |  |  |  |
| (6) vaccination | 0.09* | 0.02* | 0.01 | 0.08* | 0.11* | 1.00 |  |  |  |  |  |  |  |  |  |  |  |  |  |  |  |  |  |  |  |  |
| (7) age at marriage | 0.09* | 0.01 | 0.03* | 0.15* | 0.23* | 0.06* | 1.00 |  |  |  |  |  |  |  |  |  |  |  |  |  |  |  |  |  |  |  |
| (8) age at 1^st^ birth | 0.09* | 0.00 | 0.03* | 0.16* | 0.20* | 0.06* | (0.54)* | 1.00 |  |  |  |  |  |  |  |  |  |  |  |  |  |  |  |  |  |  |
| (9) mat education | 0.20* | 0.00 | 0.06* | 0.33* | 0.36* | 0.17* | 0.22* | 0.21* | 1.00 |  |  |  |  |  |  |  |  |  |  |  |  |  |  |  |  |  |
| (10) media exposure | 0.13* | 0.00 | 0.01 | 0.20* | 0.24* | 0.11* | 0.12* | 0.11* | 0.32* | 1.00 |  |  |  |  |  |  |  |  |  |  |  |  |  |  |  |  |
| (11) marital status | 0.02* | 0.00 | 0.04* | -0.01* | 0.03* | 0.03* | 0.00 | 0.00 | 0.05* | 0.01* | 1.00 |  |  |  |  |  |  |  |  |  |  |  |  |  |  |  |
| (12) no. children home | 0.11* | 0.00 | 0.06* | (0.55)* | 0.18* | 0.10* | 0.10* | 0.11* | 0.28* | 0.20* | -0.01 | 1.00 |  |  |  |  |  |  |  |  |  |  |  |  |  |  |
| (13) children ever born | 0.13* | 0.01 | 0.10* | (0.89)* | 0.24* | 0.11* | 0.15* | 0.17* | 0.38* | 0.24* | -0.02* | 0.70* | 1.00 |  |  |  |  |  |  |  |  |  |  |  |  |  |
| (14) contraceptive use | -0.01 | -0.02* | 0.17* | 0.00 | -0.11* | -0.05* | -0.02* | 0.01 | -0.09* | -0.10* | -0.17* | -0.04* | -0.03* | 1.00 |  |  |  |  |  |  |  |  |  |  |  |  |
| (15) mat stature | 0.22* | 0.00 | 0.00 | 0.04* | 0.06* | 0.04* | 0.06* | 0.06* | 0.13* | 0.06* | 0.02* | 0.02* | 0.04* | -0.03* | 1.00 |  |  |  |  |  |  |  |  |  |  |  |
| (16) mat BMI | -0.05* | 0.00 | 0.04* | 0.01 | -0.13* | -0.01 | -0.07* | -0.05* | -0.07* | -0.07* | -0.01 | -0.01* | 0.00 | 0.02* | -0.03* | 1.00 |  |  |  |  |  |  |  |  |  |  |
| (17) mat employment | 0.01 | 0.01 | 0.09* | 0.05* | 0.01* | 0.01 | 0.01* | 0.01* | 0.02* | 0.02* | 0.10* | 0.00 | 0.03* | 0.08* | 0.01* | -0.01* | 1.00 |  |  |  |  |  |  |  |  |  |
| (18) mat autonomy | -0.04* | 0.00 | 0.05* | 0.03* | -0.07* | -0.04* | -0.05* | -0.07* | -0.06* | -0.06* | 0.03* | -0.01* | 0.01* | 0.05* | -0.02* | 0.04* | 0.10* | 1.00 |  |  |  |  |  |  |  |  |
| (19) wealth index | -0.21* | 0.00 | -0.01* | -0.17* | -0.35* | -0.13* | -0.23* | -0.21* | -0.42* | -0.50* | -0.02* | -0.15* | -0.20* | 0.08* | -0.11* | 0.12* | -0.11* | 0.07* | 1.00 |  |  |  |  |  |  |  |
| (20) residence | 0.08* | 0.01 | 0.00 | 0.08* | 0.25* | 0.05* | 0.12* | 0.09* | 0.13* | 0.27* | -0.02* | 0.08* | 0.10* | -0.12* | 0.02* | -0.10* | 0.00 | -0.09* | -0.42* | 1.00 |  |  |  |  |  |  |
| (21) Division | -0.01* | -0.01 | 0.00 | -0.02* | -0.02* | -0.02* | 0.01* | 0.01* | -0.02* | 0.09* | 0.00 | -0.02* | -0.02* | 0.03* | -0.01* | -0.01* | 0.03* | -0.02* | -0.13* | 0.10* | 1.00 |  |  |  |  |  |
| (22) h.hold members | 0.01 | 0.00 | -0.06* | 0.07* | 0.01* | 0.02* | -0.04* | -0.02* | -0.02* | -0.01 | 0.01* | 0.25* | 0.12* | -0.08* | -0.01* | -0.01* | -0.10* | -0.12* | 0.13* | 0.04* | 0.00 | 1.00 |  |  |  |  |
| (23) h.head sex | -0.03* | 0.00 | 0.01* | 0.00 | -0.03* | -0.02* | -0.02* | -0.03* | -0.05* | -0.02* | 0.16* | -0.02* | -0.01* | -0.18* | -0.01 | 0.02* | 0.01* | 0.09* | 0.05* | 0.01* | -0.03* | -0.07* | 1.00 |  |  |  |
| (24) toilet facility | 0.16* | 0.00 | 0.00 | 0.14* | 0.28* | 0.12* | 0.16* | 0.15* | 0.33* | 0.24* | 0.02* | 0.14* | 0.17* | -0.10* | 0.08* | -0.07* | 0.02* | -0.07* | -0.45* | 0.21* | -0.04* | -0.02* | -0.05* | 1.00 |  |  |
| (25) water source | 0.04* | 0.00 | 0.00 | 0.03* | 0.06* | 0.06* | 0.02* | 0.01 | 0.05* | 0.08* | 0.00 | 0.04* | 0.04* | -0.06* | 0.00 | -0.02* | -0.01* | -0.04* | -0.07* | 0.07* | 0.08* | 0.02* | -0.01* | 0.06* | 1.00 |  |
| (26) survey year | -0.13* | -0.01 | 0.00 | -0.13* | -0.31* | -0.11* | -0.12* | -0.11* | -0.27* | -0.04* | -0.02* | -0.15* | -0.17* | 0.14* | -0.04* | 0.07* | 0.17* | 0.09* | -0.01* | -0.05* | 0.10* | -0.08* | 0.07* | -0.25* | -0.05* | 1.00 |
| ** p<0.05; correlations greater than or equal to 0.5 are shown in parentheses* | | | | | | | | | | | | | | | | | | | | | | | | | | |
